# The negative effect of antibiotics on RCC patients with immunotherapy: A systematic review and meta-analysis

**DOI:** 10.3389/fimmu.2022.1065004

**Published:** 2022-11-23

**Authors:** Zhiqiang Luo, Siyuan Hao, Yuxuan Li, Lei Cheng, Xuedong Zhou, Emine Gulsen Gunes, Shiyu Liu, Jing Chen

**Affiliations:** ^1^ State Key Laboratory of Oral Diseases, National Clinical Research for Oral Diseases, West China Hospital of Stomatology, Sichuan University, Chengdu, China; ^2^ School of Stomatology, Dalian Medical University, Dalian, China; ^3^ Department of Operative Dentistry and Endodontics, West China Hospital of Stomatology, Sichuan University, Chengdu, China; ^4^ Department of Hematologic Malignancies Translational Science, City of Hope, Duarte, CA, United States; ^5^ Key Laboratory of Oral Biomedical Research of Zhejiang Province, Stomatology Hospital, School of Stomatology, Zhejiang University School of Medicine, Zhejiang Provincial Clinical Research Center for Oral Diseases, Cancer Center of Zhejiang University, Hangzhou, China

**Keywords:** carcinoma, renal cell, antibiotics, immunotherapy, immune checkpoint inhibitors, meta-analysis

## Abstract

**Background:**

Microbiome dysbiosis is considered a predictive biomarker of clinical response in renal cell carcinoma (RCC), which can be regulated by antibiotics (ATB). Multiple studies have shown that concomitant ATB administration has inhibitory effects on immunotherapy in RCC. This review aimed to assess the impact of ATB on patient survival and tumor response in RCC with immunotherapy.

**Methods:**

Literature evaluating the effect of ATB on immunotherapy in RCC from Cochrane Library^®^, PubMed^®^, Embase^®^, Scopus^®^, and Web of Science^®^ were systematically searched. Hazard ratios (HR) for progression-free survival (PFS) and overall survival (OS), odds ratio (OR) for objective response rate (ORR) and primary progressive disease (PD) were pooled as effect sizes for clinical outcomes. Subgroup analysis was conducted to reveal the determinants of the effect of ATB on immunotherapy, including time windows of ATB exposure to immunotherapy initiation, ICIs treatment and study location. The leave-one-out approach was adopted to analyze the heterogeneity formulated. Cumulative meta-analysis adding by time was used to observe dynamic changes of the results.

**Results:**

Ten studies were included in the systematic review and six studies (with n=1,104 patients) were included in the meta-analysis, four studies were excluded for overlapping patients with subsequent larger studies and lack of unique patient-level data. ATB administration was significantly correlated with shorter PFS (HR=2.10, 95%CI [1.54; 2.85], I^2^ = 2% after omitting study Derosa et al, 2021 detected by leave-one-out approach), shorter OS (HR=1.69, 95%CI [1.34; 2.12], I^2^ = 25%) and worse ORR (OR=0.58, 95%CI [0.41; 0.84]), but no difference was observed in risk of PD (OR=1.18, 95%CI [0.97; 1.44]). No significant differences existed among the subgroups for determining the determinants of ATB inhibition.

**Conclusions:**

Concomitant ATB with immunotherapy was associated with worse PFS, OS and ORR in RCC. No publication bias was observed in this study.

**Systematic review registration:**

https://www.crd.york.ac.uk/PROSPERO/display_record.php?RecordID=349577, identifier CRD42022349577.

## 1 Introduction

Renal cell carcinoma (RCC) is one of the most severe urological cancers in both men and women, and is considered one of the most challenging cancers to diagnose and treat (1). Surgical resection is potentially curative for localized RCC, unfortunately, up to 40% of patients present with metastatic or locally advanced disease at the time of the first diagnosis (2), and approximately 20%-40% of surgically resected patients eventually relapse (3). Over the past decade, targeted agents have been recognized as the first-line treatment for metastatic renal cell carcinoma (mRCC) patients, and they commonly include vascular endothelial growth factor (VEGF), VEGF receptor inhibitors, mammalian target of rapamycin (mTOR), or tyrosine kinase inhibitor (TKI) (4, 5). In recent years, immune checkpoint inhibitors (ICIs) have unprecedently improved the treatment of patients with mRCC. Since 2015, monotherapy with nivolumab as the second-line treatment and combinations of nivolumab plus ipilimumab, pembrolizumab plus axitinib, and avelumab plus axitinib as first-line treatments have been approved for immunotherapy in patients with RCC (6). In a recent meta-analysis, ICIs were significantly more effective in prolonging overall survival (OS) (P < 0.001) and progression-free survival (PFS) (P =0.009) in patients than standard first- line VEGFR- or mTOR-targeted therapy for mRCC (7).

However, 35%-44% primary resistance to ICIs treatment in RCC patients, remains a grand challenge to clinical efficacy (8, 9). Primary resistance is not only associated with the functional exhaustion of tumor-infiltrating lymphocytes (10), immune-related adverse effects, or gene expression signatures (11); recently, the gut microbiota has become another predictive factor for the response to immunotherapy and drives primary resistance to immunotherapy in RCC patients (12–14). For example, Routy et al. used shotgun sequencing quantitative metagenomics to explore the intestinal flora of RCC patients who received ICIs treatment. They observed an increased abundance of *muciniphila*-dominated commensal bacteria in responders and found that the difference in gut microbiota composition was closely associated with prolonged PFS (14).

Due to the high risk of urinary tract and upper respiratory tract infections in RCC patients (8, 15), antibiotic agents are inevitable. As regulators of the gut microbiota, antibiotics may affect immunotherapy efficacy by altering the gut microbiota. Yang et al. observed a shorter OS and PFS existed in pooled solid tumor patients with immunotherapy and concomitant ATB in a meta-analysis (16–20). However, the effect of ATB on immunotherapy in RCC remains controversial. Some dissenting voices, including Lalani et al (21), Derosa et al (8), and Taigo et al (22) showed no statistically significant difference in the length of OS between patients ATB users and non-ATB users. Taigo et al (22) and Derosa et al (23) also did not observe significantly shorter PFS in RCC patients with ATB exposure than in non-ATB users.

As a significant number of clinical studies have been published on RCC, we aimed to conduct a systematic review and meta-analysis to answer the following questions:

Is ATB use associated with reduced efficacy of immunotherapy in RCC patients? If so, what are the key factors that determine the inhibitory effect of ATB? Time windows of ATB exposure? Line of immunotherapy? Type of ICIs treatment?

## 2 Methods

### 2.1 Protocol and search strategy

Before the initiation of this review, the protocol was pre-registered and accepted at the International Prospective Register of Systematic Reviews (PROSPERO) with ID number CRD42022349577. This review followed the Preferred Reporting Items for Systematic Reviews and Meta-Analyzes (PRISMA) statement (24). This was reported based on a systematic review of the Cochrane Handbook for Systematic Reviews.

Two investigators (Z. Luo and Y. Li) searched all relevant literature from the establishment of databases up to July 18, 2022, from Cochrane Library, PubMed, Embase, Scopus, and Web of Science according to the criteria developed together with the search keywords “Renal Cell Carcinomas,” “antibiotics,” “immunotherapy,” “ICIs,” “immune checkpoint blockage,” “programmed death 1 (PD-1),” programmed cell death-Ligand 1 (PD-L1) inhibitor,” or “cytotoxic T lymphocyte antigen-4 (CTLA-4) inhibitor”. A specific search strategy is available in the attachment (Supplementary 1) . No limitations of publication, language, region, and references of the considered studies and reviews were searched to identify any further relevant data.

### 2.2 Inclusion criteria and exclusion criteria

Comprehensive inclusion and exclusion criteria were developed for complete and accurate inclusion of relevant studies. All the selected studies were based on the inclusion and exclusion criteria.

The inclusion criteria include:

(1) All relevant randomized clinical trials, case-control, and cohort studies
(2) Data or Kaplan-Meier curves on the hazard ratio for OS or PFS.
(3) Data on objective response rate (ORR) and primary progressive disease (PD) can also be included
The exclusion criteria include:
(1) Studies with a sample size ≤ 10.
(2) Animal studies, reviews, protocols, case reports, patents, and corrections were excluded.
(3) Studies evaluating the impact of antibiotics on cancer types other than RCC or the aggregation of different cancer types.
(4) Studies covering overlapping patients reported in subsequent larger studies and lack of unique patient-level data were excluded from meta-analysis.

### 2.3 Data extraction

From each of the eligible studies, the following data were collected: basic information of literature (author, publication year, area, type of publication); information on the population, including patients (number of patients, composition of sex, and age) and cancer characteristics (histology, data on metastasis, IMDC risk); information on immunotherapy and use of ATB, including immunotherapy (line of treatment, type of immunotherapy) and antibiotic treatment (time windows of exposure to ATB compared to ICI treatment initiation, reason, duration, and type of ATB use); and information of outcomes, including median follow-up, median OS and PFS, Hazard ratios (HR) for PFS and OS, ORR, and PD.

When both univariate and multivariate analyses were reported, the results from the multivariate analysis were preferred. When HR for OS or PFS was unavailable, it was estimated from the Kaplan–Meier curves using the approach described by Tierney et al (25) and repeated calculations three times independently using the spreadsheet attached to the publication to ensure consistency of the results. Data were discarded if there was a considerable difference after multiple HR estimations.

### 2.4 Data analysis

Before initiating data analysis, we independently conducted a quality assessment of the included studies by two authors (Z. Luo and Y. Li) based on the Newcastle–Ottawa scale (26). Egger’s test (27) and funnel plots were used to analyze publication bias.

The primary outcome was the effect of antibiotic use on immunotherapy efficacy, as measured by PFS and OS in patients with RCC. HR values were used to compare PFS and OS between patients exposed to ATB and those who did not.

To identify whether different periods of antibiotic exposure would have various clinical outcomes of immunotherapy, we regrouped the studies according to the time windows of ATB exposure to immunotherapy initiation. Owing to the large differences in the exposure time windows among each study, we set out the cut-off point with the following requirements (1): To maintain a higher number of studies, cut-off points correspond to frequently reported time windows (2). different time windows of antibiotic exposure reported in the same study were considered; however, each study was included only once in each subgroup.

To meet the requirements, the following two subcategories were included in the subgroup analysis.

Group -90 to 0: Exposure to ATB 90 days before immunotherapy initiation

Group -60 to +60: Exposure to ATB between 60 days before and 60 days after immunotherapy initiation.

Subgroup analysis was also performed according to the ICIs treatment for PFS and OS, including subgroups of ICIs monotherapy vs combination therapy (two different ICIs or ICI plus target therapy) vs the mixed subgroup (mixed with ICIs monotherapy and combination therapy). Study location (USA/Europe vs East Asia) was set as another indicator of subgroup analysis for PFS.

In addition, the leave-one-out approach was applied to conduct an impact analysis of the included studies to explore the contribution of each study to the overall heterogeneity. Cumulative meta-analysis added each study in chronological order to observe the dynamic changes in the results according to the carrying-out time of the study.

To better adapt the different heterogeneity formulated in various indicators while pooling the included studies, the random effects model was used if I^2^≥50% or test of heterogeneity P ≤ 0.05, and the common effect model was used if I^2^<50% and test of heterogeneity P>0.05, to calculate pooled HR of OS and PFS and pooled OR of ORR and PD. The Knapp-Hartung method and Sidik-Jonkman estimator (28) estimated the inter-study variance, τ^2^. Higgins and Thompson I^2^ were used to measure the inter-study heterogeneity.

All analyses were performed using R version 4.2.1 (29) and the meta package (30).

## 3 Results

### 3.1 Search result

The systematic search yielded a total of 1,768 records from the Cochrane Library ^®^, PubMed^®^, Embase^®^, Scopus^®^, and Web of Science^®^, and records were manually added after viewing the references. Fifty-eight records with topic on RCC, ATB and immunotherapy were fully reviewed. Ten studies (8, 14, 15, 21–23, 31–34) were included in the systematic review, four studies (8, 14, 15, 31) were excluded from meta-analysis for the following reasons:

1. Derosa et al, 2018, Derosa et al, 2020 and Routy et al, 2018 were excluded due to the overlapping patients with subsequent larger study Derosa et al, 2021 and lack of unique patient-level data (Half of RCC patients in Derosa et al, 2018 and 40 RCC patients in Derosa et al, 2020 had been previously reported in Routy et al, 2018. Routy et al, 2018, Derosa et al, 2020 and Derosa et al, 2021 were all conducted with the patients from NIVOREN Phase II trial at Gustave Roussy between February 2016 to April 2017).

2. Kulkarni et al, 2019 was excluded due to the overlapping patients with subsequent study Kulkarni et al, 2020 and lack of unique patient-level data (Both studies were conducted with patients from M-Health Fairview health system sites in the Minneapolis region between May 2015 and December 2017).

**Figure 1:**
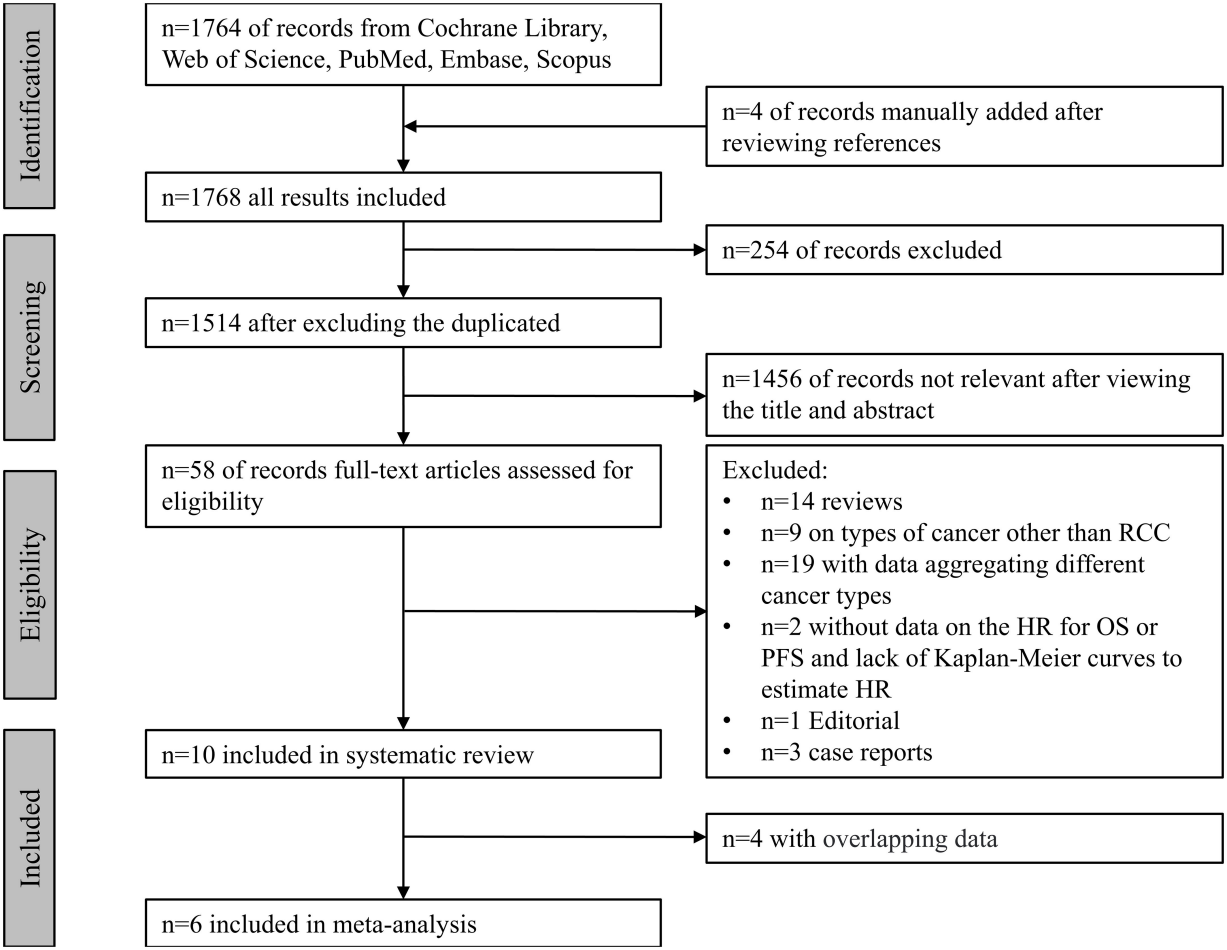
Flow diagram of included and excluded studies.

Totally, six studies (21–23, 32–34) were included for meta-analysis, with five publications and one abstract enrolling 1,104 patients for PFS, 1,073 patients for OS, 934 patients for ORR, and 788 patients for PD. A flowchart of the literature search is shown in **Figure 1**.

### 3.2 Baseline characters

The baseline characteristics of the included studies are presented in **Table 1**. (Detailed information is provided in **Supplementary Tables 1A, B**). The included studies did not show considerable heterogeneity in reported population characteristics. For population composition, patients in all studies had a male-to-female ratio of approximately 2:1, mainly with a median age of 61 to 63 years [except for the two studies in Japan, 67 and 70, respectively, in Ueda et al (32) and Taigo et al (22)]. The characteristics of RCC among the studies have a strong resemblance. All patients were diagnosed with mRCC, with renal clear cell carcinoma as the main histological type, accounting for 86.8% of all patients. Among the studies that provided IMDC risk scores, five studies from the USA and Europe have 56%-59% intermediate risk, 17.8%-22% favorable risk, and 20%-26% poor risk; however, in two studies from Japan, we observed an over 90% proportion of intermediate and poor risk in RCC patients. Lung metastasis was the main form of RCC metastasis in all studies, followed by bone, brain, liver, and lymph node metastases.

**Table 1:** Basic characteristics of included studies.

Basic information			Time window of ABT exposure to ICI initiation	ICIs treatment	Line of treatment	Outcomes						
Author,year	Area	Number of patients				mFollow-up(range)(months)	mPFSATB+ vs. ATB-Δ(months)	mOSATB+ vs. ATB-Δ (months)	HR for PFS (95% CI)	HR for OS (95% CI)	ORRATB+ vs. ATB-	PDATB +vs. ATB-
**UEDA et al,** **2019 (** 32)	Japan	n=31(in total) n=5(29.9% with ATBs)	-30d to 0	nivolumab: 90.3% Ipilimumab + nivolumab: 9.7%	first-line:9.7% second- line:90.3%	NA	2.8 vs.18.4 Δ=15.6	NA	(Univariate) 6.518(1.857-21.416) (Multivariate) 3.830 (1.086-12.717)	not affected	not affected.	NA
**Lalani et al,** **2020 (** 21)	the USA	n=146(in total) n=31(21% with ATBs)	-8w to +4w	anti-PD-(L)1–monotherapy:54.8% anti-PD-(L)1–combination:44.2%	first-line:43.2% second- line:26.7% third-line or later:30.1%	16.6 (0.7–67.8)	2.6vs.8.1 Δ=4.5	NA	(Multivariate) 1.96(1.20–3.20)	(Multivariate) 1.44 (0.75–2.77)	12.9% vs.34.8% p=0.026	NA
		n=146(in total) n=27(18.5% with ATBs)	-30d to +30d	anti-PD-(L)1–monotherapy:54.8% anti-PD-(L)1–combination:44.2%	first-line:43.2% second- line:26.7% third-line or later:30.1%	16.6 (0.7–67.8)	NA	NA	(Multivariate) 2.03 (1.21–3.41)	(Multivariate) 1.59 (0.80–3.15)	NA	NA
**Kulkarni et al,** **2020 (** 33)	the USA	n=55(in total) n=24(44% with ATBs)	-1m to +6w	nivolumab: 93% others: 7% (monotherapy)	first-line:7% second-line or later:93%	18.7	2.7 vs 4.2 Δ=1.5	17 vs 22 Δ=5	(Multivariate) 2.7(1.3-5.9)	(Multivariate) 4.2(1.5-12.2)	NA	NA
**Guven et al,** **2021 (** 34)	Turkey	n=93(in total) n=31(33.3% with ATBs)	-3m to +3m	nivolumab and others	second- line:54.8% third-line or later:45.2%	10.87	NA	NA	(Multivariate) 2.238 (1.284-3.900)	(Multivariate) 2.306 (1.155-4.601)	24.1% vs.50%, P=0.023	41.4% vs.23.1% P=0.084
**Derosa et al,** **2021 (** 23)	France	n=707(in total) n=104(14.7% with ATBs)	-60d to +42d	nivolumab (monotherapy)	NA	NA	2.6 vs.3.8 Δ=1.2	13.0 vs.25.0 Δ=12	(Univariate) 1.24 (0.99-1.55)	(Univariate) 1.77 (1.36-2.31) (Multivariate) 1.59 (1.22-2.09)	15.1vs.21.1%, P=0.176	57% vs. 47.3%
**Taigo Kato et al,2022 (** 22)	Japan	n=72(in total) n=47(65.3% with ATBs)	-3m to 0	nivolumab plus ipilimumab:100% (combination therapy)	first-line:100%	16.1 (1.4–37.8)	13.2 vs. NR	NA	(Univariate) 0.86(0.29-2.53)	(Univariate) 0.66(0.13-3.35)	NA	NA
**Derosa et al,** **2018 (** 8)	France	n=121(in total) n=16(13% with ATBs)	-30d to 0	anti-PD-(L)1 therapy: 88% anti-PD-(L)1+CTLA-4: 8% anti-PD-(L)1 + bevacizumab: 4%	first-line:57% second-line or later:43%	NA	1.9 vs 7.4 Δ=5.5	17.3 vs.30.6 Δ=13.3	(Univariate) 3.1 (1.4-6.9) (Multivariate) 2.2 (1.3–3.3)	(Univariate) 3.5 (1.1-10.8) (Multivariate) 2.1 (0.9–5.0)	13% vs.26%	75% vs.22% P < 0.01
		n=121(in total) n=22(18% with ATBs)	-60d to 0	anti-PD-(L)1 therapy: 88% anti-PD-(L)1+CTLA-4: 8% anti-PD-(L)1 + bevacizumab: 4%	first-line:57% second-line or later:43%	NA	3.1 vs 7.4 Δ=4.3	23.4 vs.30.0 Δ=6.6	(Univariate) 1.9 (1.1-3.1) (Multivariate) 3.2 (1.6-5.9)	(Univariate) 2.0 (0.9-4.3)	18% vs.25%	64% vs.21% P < 0.01
**Routy et al,** **2018 (** 14)	multicenter	n=67(in total) n=20(29.9% with ATBs)	-2m to +1m	anti-PD-1: 92% anti-PD-L1: 8%	NA	NA	4.3 vs 7.4 Δ=3.1	23.4 vs 27.9 Δ=4.5	(Univariate) 2.16 (1.18-3.96) (Multivariate) 2.12 (1.11–4.05)	(Univariate) 1.22(0.84–1.91)	NA	NA
**Kulkarni et al,** **2019 (** 31)	the USA	n=55(in total) n=40(72% with ATBs)	-< 1m to during	anti-PD-(L)1	NA	NA	2.9 v 5.0 Δ=2.1	not affected.	(Univariate) 2.3(1.0-5.0)	not affected	not affected.	NA
**Derosa et al,** **2020 (** 15)	France	n=69(in total) n=11(16% with ATBs)	NA	nivolumab	NA	23.54 (0.66-32.21)	1.87 vs 5.09 Δ=3.22	24.6 vs undefined	(Univariate) 3.85(1.69–8.78)	(Univariate) 3.84 (1.16–12.70)	9% vs.28% P< 0.03	73% vs.33%

ATB, antibiotics; ATB+, antibiotics present; ATB, antibiotics absent; w, week(s); m, month(s); d, days; NA, not available; NR, not reached; mFollow-up, median follow-up; mPFS, median progression free survival; mOS, median overall survival; OS, overall survival; PFS, progression-free survival; HR, hazard ratio; CI, confidence interval; ORR, objective response rate; PD, primary progressive disease; P, p-value.

The baseline immunotherapy and antibiotic use varied among the studies. PD-1/PD-L1 monoclonal antibody administration was the most common, either monotherapy (mostly nivolumab, as second-line treatment) or combined therapy with a VEGF inhibitor (bevacizumab) and anti-CTLA-4 monoclonal antibody (ipilimumab). The line of therapy showed considerable heterogeneity between studies; the proportion of first-line treatment ranged from 7% to 100%. The reason, type, duration of antibiotic use, and the time windows of ATB exposure varied considerably across the included cohorts. The time windows of antibiotic exposure relative to ICIs initiation have been provided in all studies and were considered an essential factor in the outcome. Only two studies, Derosa et al, 2018 (8) and Derosa et al, 2020 (15), carefully reported the reasons for using ATB, among which urinary tract infection was the main indication, followed by flu-like syndrome, mTOR inhibitor-associated events and perioperative use. For ATB types, β-lactam ± inhibitors were used most frequently, followed by quinolones (However, in Guven et al, 2021 (34), quinolones were the most frequently used antibiotics, accounted for 48.4%). Only Kulkarni et al, 2020 (33) reported the influence of antibiotic type on the clinical effect of ICIs treatment. The duration of ATB was grouped into short-term (≤7 days) and long-term (> 7 days) use, which did not show significant differences between the studies.

### 3.3 Effects of ATB on PFS

#### 3.3.1 Effects of ATB on overall PFS

**Figure 2:**
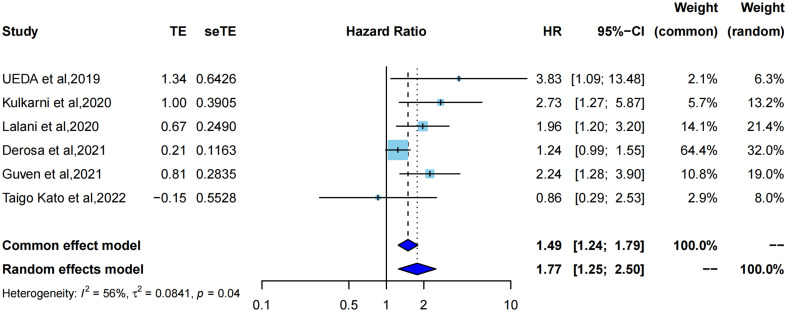
Forest plot of hazard ratios for progression free survival of RCC patients exposed to antibiotics versus not exposed to antibiotics around immunotherapy.

The HR for PFS was extracted from six studies (21–23, 32–34) for meta-analysis, and one study was assessed using Kaplan–Meier curves. ATB exposure was significantly correlated with worse PFS in RCC patients treated with ICIs (pooled HR =1.77, 95%CI [1.25; 2.50]) by a random-effects model (I^2^ = 56%, P=0.04) (**Figure 2**). Correspondingly, the data of each study also indicated that median PFS had different degrees of reduction, ranging from 1.2 months to 15.6 months, and the average reduction was 5.7 months. Cumulative meta-analysis was also used to identify dynamic changes caused by the inclusion of different studies by time order. (**Supplementary Figure 1**). With the addition by time order, the 95% confidence interval of HR was narrowed from pooled HR=3.83, 95%CI [1.09; 13.48] in 2019 to pooled HR=1.77, 95%CI [1.25; 2.50] in 2022; therefore, the effects of ATB exposure on PFS could be more objectively and accurately reflected. Additionally, I^2^ had a noticeable change from 0% to 64% after adding Derosa et al, 2021.

#### 3.3.2 Subgroup analysis for effects of ATB on PFS

According to the time windows of ATB exposure relative to ICIs initiation, there was a significant correlation with shorter PFS and ATB exposure in Group -60 - +60 subgroup (pooled HR=1.86, 95%CI [1.18; 2.95], with I^2^ = 64% by random effects model),but not Group -90 – 0 subgroup (pooled HR=1.75, 95%CI [0.40; 7.55], with I^2^ = 68% by random effects model). Unfortunately, no significant difference was found between the two groups (p=0.93) (**Supplementary Figure 2**).

On subgroup analysis in terms of ICIs treatment, there was a significant correlation with shorter PFS and ATB exposure in the mixed subgroup (pooled HR=2.18, 95%CI [1.53; 3.10]), not the monotherapy subgroup (pooled HR=1.68, 95%CI [0.79; 3.59], I^2^ = 73%) or combination therapy (HR=0.86, 95%CI [0.29; 2.53]). No significant difference was observed between the subgroups (P=0.25) (**Supplementary Figure 3**).

On the subgroup analysis of study location, by the random effects model, there was a significant correlation with shorter PFS and ATB exposure in the USA or Europe subgroup (HR=1.79, 95%CI [1.23; 2.60] with I^2^ = 64% and P=0.04), not the East Asia (HR=1.75, 95%CI [0.40; 7.55] with I^2^ = 68% and P=0.08). No significant difference was observed between the subgroups (P=0.98) (**Supplementary Figure 4**).

### 3.4 Effects of ATB on OS

#### 3.4.1 Effects of ATB on OS

**Figure 3:**
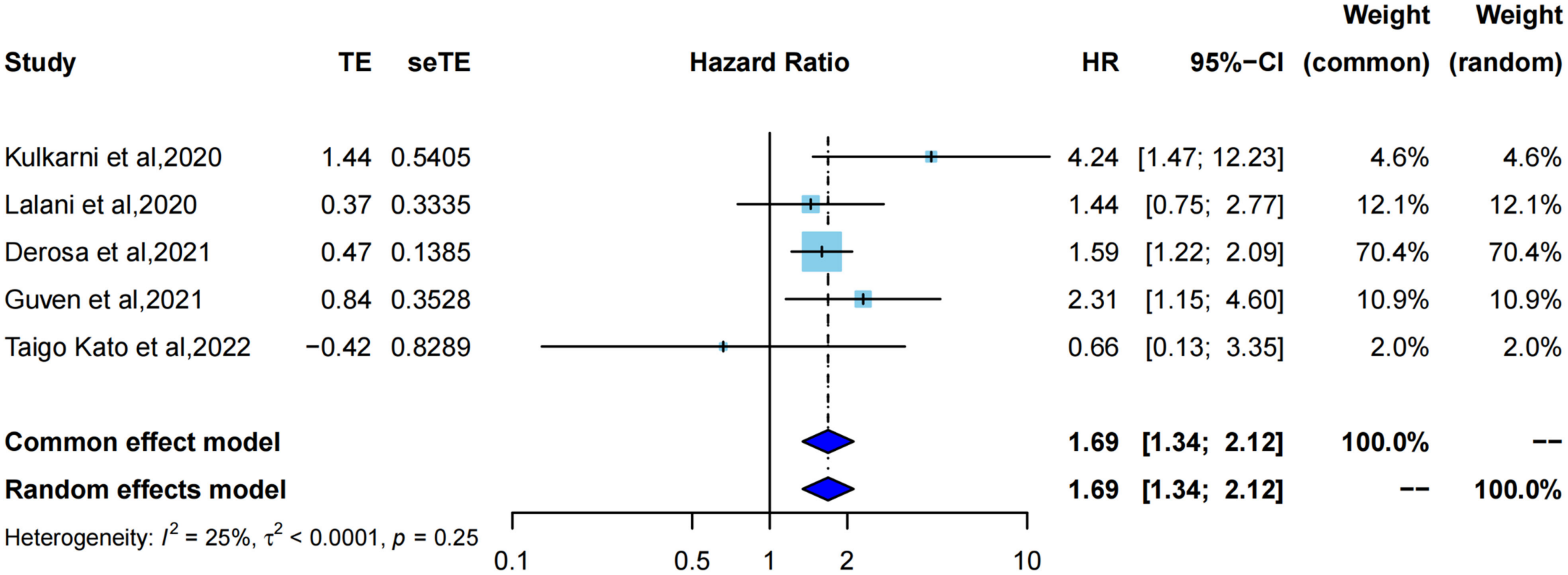
Forest plot of hazard ratios for overall survival of RCC patients exposed to antibiotics versus not exposed to antibiotics around immunotherapy.

HR for OS was extracted from five studies (21–23, 33, 34) for meta-analysis, and one study was assessed using Kaplan–Meier curves. As shown in (**Figure 3**), using the common effect model (I^2^ = 25%, P=0.25), ATB exposure showed a significantly lower OS than no ATB exposure (pooled HR=1.69, 95%CI [1.34; 2.12]). Correspondingly, the median OS in each study also showed different degrees of reduction, ranging from 5 months to 12 months, with a mean reduction of 8.5 months.

Cumulative meta-analysis (**Supplementary Figure 5**) revealed that with the inclusion of studies by time order, the length of the 95% confidence interval was constantly shortened (to 2020, HR=4.24, 95%CI [1.47; 12.23]) to statistical significance (to 2022, pooled HR=1.69, 95%CI [1.34; 2.12]), which also indicated that the pooled results tended to be more objective, accurate, and close to the truth.

#### 3.4.2 Subgroup analysis for effects of ATB on OS

According to the time windows of ATB exposure relative to ICIs initiation, there was a significant correlation with shorter OS in the group -60 +60 ATB exposure subgroup by the common effect model (pooled HR=1.66, 95%CI [1.30; 2.11], I^2^ = 39%, P=0.19), not the group -90 to 0 subgroup (HR=0.66, 95%CI [0.13; 3.35]). No significant differences were observed between the two groups (p=0.27) (**Supplementary Figure 6**).

On subgroup analysis in terms of ICIs treatment, there was a significant correlation with shorter OS in the mixed subgroup (HR=1.80, 95%CI [1.12; 2.89] with I^2^ = 0% and P=0.33), not the monotherapy subgroup (pooled HR=2.26, 95%CI [0.90; 5.67] with I^2^ = 67% and P=0.08) and combination therapy (HR=0.66, 95%CI [0.13; 3.35]). No significant difference was observed between the subgroups (P=0.43) (**Supplementary Figure 7**).

### 3.5 Sensitivity analysis of OS and PFS

**Figure 4:**
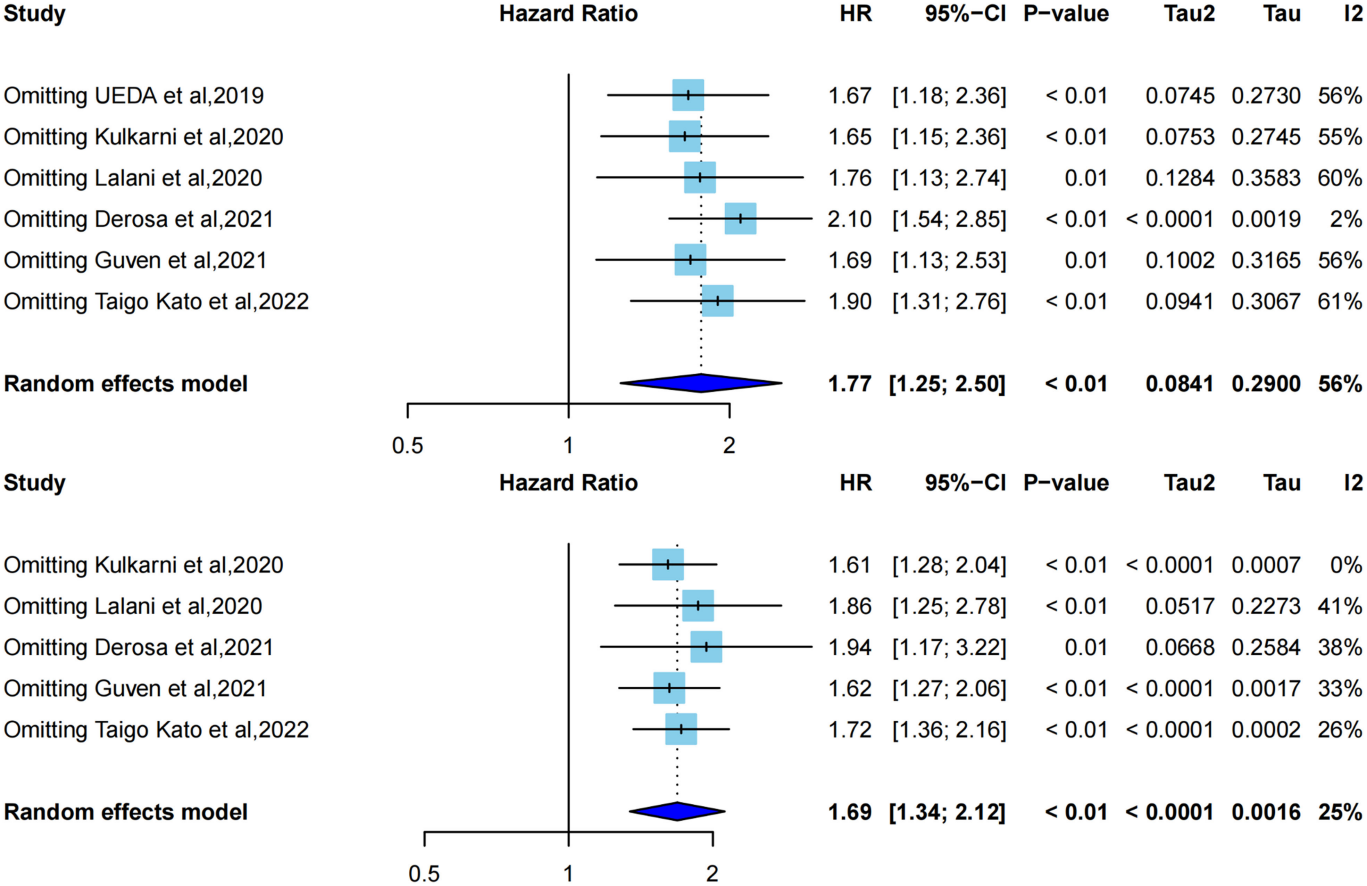
Influence analysis representing the influence of each included study on the pooled HR and overall heterogeneity for OS and PFS. **A**, leave-one-out analysis for PFS; **B**, leave-one-out analysis for OS.

**Figure 5:**
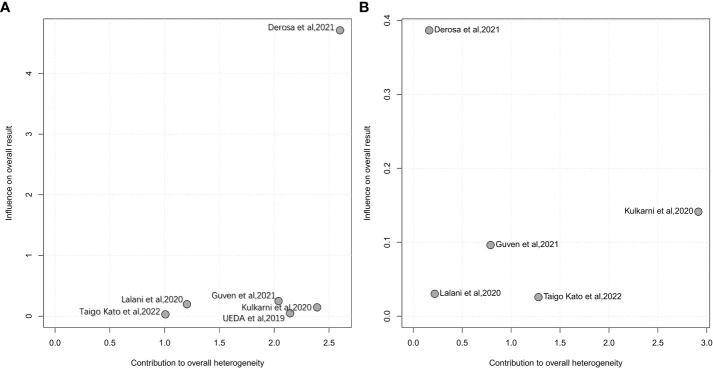
baujat plot of influence analysis. **A**, baujat plot for PFS; **B**, baujat plot for OS.

Influence analysis using a leave-one-out approach to explore the heterogeneity formulation represented the influence of each included study on the pooled HR and overall heterogeneity for PFS and OS (**Figures 4**, **5**). For PFS, Derosa et al, 2021 (23) contributed the most to the heterogeneity and the result. The pooled HR of PFS was significantly different after omitting Derosa et al, 2021 (23) in the sensitivity analysis (HR=2.10, 95%CI [1.54, 2.85] with no heterogeneity (I^2^ = 2%). However, after omitting any other studies, the results maintained an I^2^ ranging from 55% to 61%. This result illustrated that the Derosa et al, 2021 (23) study was the major source of heterogeneity formulation. The Baujat plot (**Figure 5A**) also pointed this out.

Cumulative meta-analysis (**Supplementary Figure 1**), studies before 2020 showed no heterogeneity (I^2^ = 0%), and heterogeneity directly increased to I^2^ = 64% after the inclusion of Derosa et al, 2021 (23). The heterogeneity did not change much following the addition of the next two studies [Guven et al, 2021 (34) and Taigo Kato et al, 2022 (22)].

### 3.6 Effects of ATB on ORR and PD

**Figure 6:**
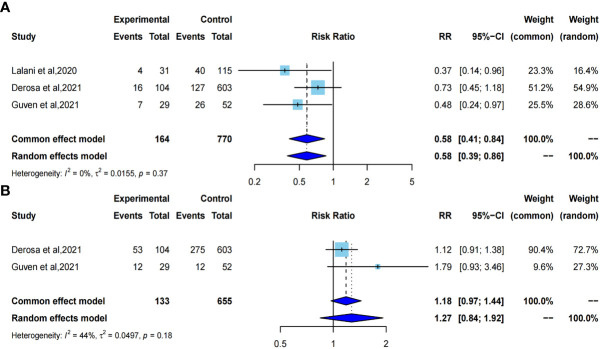
Forest plot of odds ratios for objective response rate and primary progressive disease. **A**, odds ratio for objective response rate; **B**, odds ratio for primary progressive disease.

We selected the odds ratio as the effect size to evaluate the effect of ATB exposure on ORR and PD in RCC patients undergoing immunotherapy. Pooled results showed that ATB exposure compared with no ATB exposure was significantly correlated with a worse ORR in cancer patients treated by ICIs (pooled OR=0.58, 95%CI [0.41; 0.84]) with no heterogeneity using a common-effect model (I^2^ = 0%, P=0. 37). However there was no significant correlation with increased risk of PD between ATB exposure and ATB absent (pooled OR=1.18, 95%CI [0.97;1.44]) using a common-effect model (I^2^ = 44%, P =0.18) (**Figure 6**).

### 3.7 Publication bias and quality evaluation

**Figure 7:**
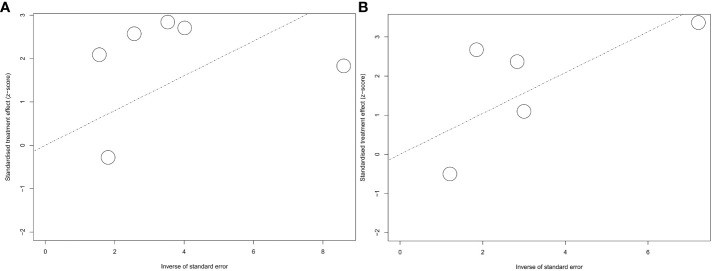
Funnel plot for hazard ratios for PFS and OS. **A**, funnel plot for PFS; **B**, funnel plot for OS.

The Newcastle–Ottawa scale was used for the quality evaluation. The included studies were scored from 5 to 9 (**Supplementary Table 2**) indicated that all the studies were eligible for the systematic review and meta-analysis. Egger’s test was used to quantify the publication bias for PFS and OS. The results were presented as the funnel plots (**Figure 7**).

No publication bias was shown by the Egger’s test (P=0.1598) for PFS. However, we still observed a lack of publications in the upper-right corner of the funnel plot of PFS, suggesting a lack of positive effects of ATB on immunotherapy efficacy (**Figure 7A**). For OS, no publication bias was indicated by Egger’s test (P=0.7350) with a basically symmetric funnel plot (**Figure 7B**).

## 4 Discussion

Although the results of various studies were controversial, the pooled results of our study still indicated shorter PFS with pooled HR=1.77, 95%CI [1.25; 2.50] [pooled HR=2.10, 95%CI [1.54; 2.85] after omitting Derosa et al, 2021 (23)], shorter OS with pooled HR=1.69, 95%CI [1.34; 2.12], lower ORR with pooled OR=0.58, 95%CI [0.41; 0.84], but similar increased risk of PD with pooled OR=1.18, 95%CI [0.97; 1.44] with ATB exposure compared to no-ATB, which indicates that ATB may be a negative prognostic factor for RCC with immunotherapy. Interestingly, these four indicators of survival and tumor response showed different heterogeneities. No heterogeneity was observed in OS, ORR and PD (I^2^ = 25%, I^2^ = 0% and 44%, respectively). Conversely, significant heterogeneity was observed in PFS (I^2^ = 56%). Therefore, we used a variety of feasible analysis methods to explore the sources of the formulated heterogeneity for PFS. Owing to the limited data, we performed subgroup analyses based only on the time windows of ATB exposure, ICIs treatment and study location. Unfortunately, we didn’t identify the origin of heterogeneity on subgroup analyses. And we also adopted the leave-one-out approach to conduct impact analysis, and it was shown that Derosa et al, 2021 (23) was the major source of formulated heterogeneity. Derosa et al, 2021 (23) contributed the most to the result and the overall heterogeneity, which might due to the imperfect methodology of the abstract literature itself.

Despite evidence of the inhibitory effect of ATB on immunotherapy that has been found in several meta-analyses and systematic reviews with a solid tumor population (16–19, 35), it is inevitable that differences in solid tumor types lead to the heterogeneity among studies (17, 19). Moreover, the results of a unified analysis covering multiple solid tumors are also lacking for guidance on the accuracy of solid tumor immunotherapy and antibiotic administration. Therefore, a meta-analysis of single solid tumor patients as the only population is particularly essential. Lurienne et al (36) analyzed the effect of ATB on the efficacy of immunotherapy in patients with non-small cell lung cancer (NSCLC) as a single population. With the increasing number of studies related to RCC, we conducted a comprehensive summary of all the findings in this field from 2018 to the present. Interestingly, in contrast to Lurienne et al (36), with high heterogeneity in NSCLC patients, we only observed moderate heterogeneity in PFS in RCC patients. Moreover, only heterogeneity was also identified by influence analysis, which means that our pooled results were closer to the truth and possessed higher confidence in focusing on RCC. After a rigorous search, this study is the first systematic review and meta-analysis to assess the impact of antibiotics on survival in RCC patients with immunotherapy. It is also the most extensive list of studies on this topic from 2018 to date, including publications and abstracts. The Egger’s test showed no significant publication bias in either PFS or OS.

ATB treatment can primarily lead to the modification of the human microbiota, especially the gut flora. *Akkermansia muciniphila* is the species most strongly associated with good clinical outcomes in RCC patients with immunotherapy for its function of recruiting CD4^+^ cells and dendritic cells (37). And some species such as *Bacteroides*, *Firmicutes*, and *Bifidobacterium* can also increase the response to immunotherapy and are associated with better prognosis. In contrast, species such as *Clostridium hathewayi* and *Eggerthelia lenta* were observed with a higher relative abundance in non-responders to immunotherapy in RCC patients and were associated with worse prognosis (14, 15, 38). Although, gut microbiota serves as a double-edged sword for cancer immunotherapy. At least, the homeostasis between host immunity and gut microbes is greatly challenged under ATB treatment, especially the broad range ATB. In addition, several studies have indicated that antibiotics may directly affect the effectiveness of immunotherapy. On the one hand, the inherent anti-inflammatory effects of ATB, such as quinolone drugs, can reduce the levels of pro-inflammatory cytokines, and macrolide drugs can reduce T cell responses and thereby have a potential antagonistic effect on ICIs (35). On the other hand, some ATB-like metronidazoles can induce DNA damage and have potential carcinogenic effect (39).The interaction mechanism of the ATBs and the ICIs in RCC patients needs to be elucidated by future studies.

Drug-drug interactions are an inevitable issue in cancer treatment, which may potentially affect the clinical outcome of cancer patients. With the widespread use of immunotherapy in renal cell cancer, in addition to ATB, some other concomitant drugs with ICIs are drawing more and more attention. PPI, as another regulator of intestinal microbiota, PPI induced dysregulation of intestinal microbiota may be a factor impacting the effect of ICIs. Yin et al. observed that chronic use of PPI was associated with increased incidence of ICIs relevant colitis (40). However, Santoni et al. observed no difference revealed on ORR, PFS, OS or overall clinical benefit in RCC patients with immunotherapy between PPI exposure and PPI absent (41, 42). Interestingly, unlike the inhibitory effect of antibiotics, concomitant Statin with ICIs showed a positive effect on advanced RCC patients in the prolonged OS and PFS and higher overall clinical benefit (41, 43).

The main limitation of this study is the lack of data. First, half of RCC patients in Derosa et al, 2018 and 40 RCC patients in Derosa et al, 2020 have been previously reported in Routy et al, 2018. Routy et al, 2018, Derosa et al, 2020 and Derosa et al, 2021 were both conducted with the patients from NIVOREN Phase II trial at Gustave Roussy between February 2016 to April 2017. Both Kulkarni et al, 2019 and Kulkarni et al, 2020 were conducted with patients in M-Health Fairview health system sites in the Minneapolis region between May 2015 and December 2017. Therefore, we only kept Derosa et al, 2021 and Kulkarni et al, 2020 and excluded the other four studies (8, 14, 15, 31) in order to avoid artificially inflate the combined statistical power of the overlapping cases, finally, only six studies (n=1,104 patients) were included in meta-analysis. Second, although we got some interesting results from subgroup analysis that there was no significant correlation with shorter PFS or OS and ATB exposure in the subgroups of monotherapy, combination therapy and Group -90 - 0, which implies the disappearance of antibiotic inhibition, however, due to the limited number of included studies, those conclusions still need to be further verified by more, larger, well-conducted prospective studies. Third, we have made a lot of effort into data collection, however there are still some data of potentially key factors that are not available, including clinical outcomes on different types of ICIs (anti-PD-(L)1 vs CTLA-4), therapy mode (anti-PD(L)1 monotherapy vs combination with other target therapy), line of treatment (first line vs later), type of ATB (β-lactam ± inhibitors vs quinolones vs others), duration of ATB, IMDC risk(good vs intermediate vs poor), treatment exposure, cancer histology (sarcomatoid differentiation or not), and type of infection (urinary infection vs others), which prevented us from conducting separate subgroup analyses of these factors, hindering further investigation. Another limitation is the retrospective nature of the studies included in the analysis. First, retrospective studies did not provide effective control for irrelevant variables, including age, sex, concomitant drugs, comorbidities, or other medical variables. Therefore, the HR from the multivariate analysis was preferred to offset the influence of these confounding factors on the result, expected to consider ATB use as an independent risk factor. Second, the retrospective nature of the study inevitably led to a particular recall bias. Unlike OS, which is regarded as a reliable variable, the evaluation of PD, ORR, or PFS could be biased by the investigators. Additionally, although Derosa et al, 2021 (23) has been proven to be the major source of pooled HR of PFS heterogeneity by influence analysis, due to the limited information of abstract literature itself, it is regrettable that we could not obtain the specific methodological characteristics of this study and conduct a comprehensive analysis to explore the specific reasons for the formation of heterogeneity.

In conclusion, this study showed that concomitant antibiotic therapy with immunotherapy is associated with decreased survival and worse tumor response in RCC. No significant differences existed among the subgroups. Time windows of ATB exposure and ICIs treatment might be key factors determining the inhibitory effect of ATB. The use of concomitant ATB seems inevitable because RCC patients are highly susceptible to infection. Therefore, this meta-analysis highlights the need for more, larger, well-conducted prospective studies evaluating patients’ survival and changes in the microbiota to further explore the key factors determining the effects of ATB to seek the best combination of common ATB and immunotherapy, thus ameliorating the suppression of immunotherapy by ATB. In addition, studies have also indicated that microbiome dysregulation is a predictive biomarker of clinical response in many other malignant tumors, including oral squamous cell carcinoma (44, 45) and melanoma (46, 47); however, the inhibitory effect of ATB on immunotherapy also remains controversial (48–50). Therefore, there is a need for more relevant research on these solid tumors. Only with more robust data will we be able to provide the best guidance on the use of antibiotics while receiving immunotherapy for advanced renal cell carcinoma patients.

## Data availability statement

The original contributions presented in the study are included in the article/**Supplementary Material**. Further inquiries can be directed to the corresponding authors.

## Author contributions

Author contributions: JC and ZL: conception and design, acquisition of data, analysis and interpretation of data, statistical analyses, and drafting and revising of the manuscript. SH carried out the data extraction and quality assessment, analyzed the data, and drafted the manuscript. YL: acquisition of data, analysis and interpretation of data, and critical revision of the manuscript. SL, LC, XZ, and EG analyzed and interpreted the data and critically revised the manuscript. All authors contributed to the article and approved the submitted version.

## Funding

This work was supported by the Research Funding from West China School/Hospital of Stomatology Sichuan University, No. RCDWJS2022-5 (to JC), and the fellowship of China Postdoctoral Science Foundation 2022M712239 (to JC).

## Conflict of interest

The authors declare that the research was conducted in the absence of any commercial or financial relationships that could be construed as a potential conflict of interest.

## Publisher’s note

All claims expressed in this article are solely those of the authors and do not necessarily represent those of their affiliated organizations, or those of the publisher, the editors and the reviewers. Any product that may be evaluated in this article, or claim that may be made by its manufacturer, is not guaranteed or endorsed by the publisher.

## Abbreviations

RCC, renal cell carcinoma; mRCC, metastatic renal cell carcinoma; HR, hazard ratio; OR, odds ratio; OS, overall survival; PFS, progression-free survival; ORR, objective response rate; PD, primary progressive disease; VEGF, Vascular Endothelial Growth Factor; mTOR, mammalian target of rapamycin; TKI, Tyrosine Kinase Inhibitor; ATB, antibiotics; PD-1, programmed death 1; PD-L1, programmed cell death-Ligand 1; CTLA-4, Cytotoxic T Lymphocyte Antigen-4; IMDC, International Metastatic RCC Database Consortium; NSCLC, non-small cell lung cancer; PPI, Proton Pump Inhibitors.
