# Supplementary material for: The negative effect of antibiotics on RCC patients with immunotherapy: A systematic review and meta-analysis
Source: Front Immunol. 2022 Nov 23;13:1065004. doi: 10.3389/fimmu.2022.1065004 (PMC9727164; doi:10.3389/fimmu.2022.1065004)

Supplementary materials:

Supplementary 1. Search strategy.

Supplementary table 1a and 1b. Detailed information of basic characteristics of included studies.

Supplementary table 2. Study quality of included studies based on the Newcastle-Ottawa scale

Supplementary figure 1. Forest plot of cumulative meta-analysis of hazard ratios for progression free survival of RCC patients, adding by the order of year.

Supplementary figure 2. Forest plot of subgroup analysis of hazard ratios for progression-free survival, according to the time window of antibiotic exposure.

Supplementary figure 3. Forest plot of subgroup analysis of hazard ratios for progression-free survival of RCC patients exposed to antibiotics versus not exposed to antibiotics around immunotherapy, according to the ICIs treatment.

Supplementary figure 4. Forest plot of subgroup analysis of hazard ratios for progression-free survival, according to the study location.

Supplementary figure 5. Forest plot of cumulative meta-analysis of hazard ratios for overall survival of RCC patients, adding by the order of year.

Supplementary figure 6. Forest plot of subgroup analysis of hazard ratios for overall survival, according to the time window of antibiotic exposure.

Supplementary figure 7. Forest plot of subgroup analysis of hazard ratios for overall survival according to ICIs treatment.

Supplementary 1. Search strategy

PubMed search strategy：

(("Immune Checkpoint Inhibitors"[Mesh]) OR (("Immunotherapy"[All Fields]) OR ("immunological therapy"[All Fields]) OR ("immunotherapies"[All Fields]) OR ("Immune Checkpoint Inhibitors"[All Fields]) OR ("Checkpoint Inhibitors, Immune"[All Fields]) OR ("Immune Checkpoint Inhibitor"[All Fields]) OR ("Checkpoint Inhibitor, Immune"[All Fields]) OR ("Immune Checkpoint Blockers"[All Fields]) OR ("Checkpoint Blockers, Immune"[All Fields]) OR ("Immune Checkpoint Blockade"[All Fields]) OR ("Checkpoint Blockade, Immune"[All Fields]) OR ("Immune Checkpoint Inhibition"[All Fields]) OR ("Checkpoint Inhibition, Immune"[All Fields]) OR ("PD-L1 Inhibitors"[All Fields]) OR ("PD L1 Inhibitors"[All Fields]) OR ("PD-L1 Inhibitor"[All Fields]) OR ("PD L1 Inhibitor"[All Fields]) OR ("Programmed Death-Ligand 1 Inhibitors"[All Fields]) OR ("Programmed Death Ligand 1 Inhibitors"[All Fields]) OR ("PD-1-PD-L1 Blockade"[All Fields]) OR ("Blockade, PD-1-PD-L1"[All Fields]) OR ("PD 1 PD L1 Blockade"[All Fields]) OR ("CTLA-4 Inhibitors"[All Fields]) OR ("CTLA 4 Inhibitors"[All Fields]) OR ("CTLA-4 Inhibitor"[All Fields]) OR ("CTLA 4 Inhibitor"[All Fields]) OR ("Cytotoxic T-Lymphocyte-Associated Protein 4 Inhibitors"[All Fields]) OR ("Cytotoxic T Lymphocyte Associated Protein 4 Inhibitors"[All Fields]) OR ("Cytotoxic T-Lymphocyte-Associated Protein 4 Inhibitor"[All Fields]) OR ("Cytotoxic T Lymphocyte Associated Protein 4 Inhibitor"[All Fields]) OR ("PD-1 Inhibitors"[All Fields]) OR ("PD 1 Inhibitors"[All Fields]) OR ("PD-1 Inhibitor"[All Fields]) OR ("Inhibitor, PD-1"[All Fields]) OR ("PD 1 Inhibitor"[All Fields]) OR ("Programmed Cell Death Protein 1 Inhibitor"[All Fields]) OR ("Programmed Cell Death Protein 1 Inhibitors"[All Fields]) OR ("Abatacept"[All Fields]) OR ("atezolizumab"[All Fields]) OR ("Ipilimumab"[All Fields]) OR ("Nivolumab"[All Fields]) OR ("pembrolizumab"[All Fields]) OR ("relatlimab "[All Fields]) OR ("sotorasib"[All Fields]) OR ("spartalizumab"[All Fields]))) AND ((("Carcinoma, Renal Cell"[Mesh]) OR

(("Carcinoma, Renal Cell"[All Fields]) OR ("Carcinomas, Renal Cell"[All Fields]) OR ("Renal Cell Carcinomas"[All Fields]) OR ("Nephroid Carcinoma"[All Fields]) OR ("Carcinoma, Nephroid"[All Fields]) OR ("Nephroid Carcinomas"[All Fields]) OR ("Adenocarcinoma Of Kidney"[All Fields]) OR ("Adenocarcinoma Of Kidneys"[All Fields]) OR ("Kidney, Adenocarcinoma Of"[All Fields]) OR ("Renal Cell Carcinoma"[All Fields]) OR ("Renal Cell Cancer"[All Fields]) OR ("Cancer, Renal Cell"[All Fields]) OR ("Renal Cell Cancers"[All Fields]) OR ("Adenocarcinoma, Renal"[All Fields]) OR ("Renal Adenocarcinoma"[All Fields]) OR ("Renal Adenocarcinomas"[All Fields]) OR ("Renal Carcinoma"[All Fields]) OR ("Carcinoma, Renal"[All Fields]) OR ("Renal Carcinomas"[All Fields]) OR ("Adenocarcinoma, Renal Cell"[All Fields]) OR ("Adenocarcinomas, Renal Cell"[All Fields]) OR ("Renal Cell Adenocarcinoma"[All Fields]) OR ("Renal Cell Adenocarcinomas"[All Fields]) OR ("Chromophobe Renal Cell Carcinoma"[All Fields]) OR ("Sarcomatoid Renal Cell Carcinoma"[All Fields]) OR ("Papillary Renal Cell Carcinoma"[All Fields]) OR ("Renal Cell Carcinoma, Papillary"[All Fields]) OR ("Chromophil Renal Cell Carcinoma"[All Fields]) OR ("Clear Cell Renal Cell Carcinoma"[All Fields]) OR ("Grawitz Tumor"[All Fields]) OR ("Tumor, Grawitz"[All Fields]) OR ("Clear Cell Renal Carcinoma"[All Fields]) OR ("Carcinoma, Hypernephroid"[All Fields]) OR ("Hypernephroid Carcinoma"[All Fields]) OR ("Hypernephroid Carcinomas"[All Fields]) OR ("Hypernephroma"[All Fields]) OR ("Hypernephromas"[All Fields]) OR ("Collecting Duct Carcinoma (Kidney)"[All Fields]) OR ("Carcinoma, Collecting Duct (Kidney)"[All Fields]) OR ("Carcinomas, Collecting Duct (Kidney)"[All Fields]) OR ("Collecting Duct Carcinomas (Kidney)"[All Fields]) OR ("Collecting Duct Carcinoma of the Kidney"[All Fields]) OR ("Renal Collecting Duct Carcinoma"[All Fields]) OR ("Collecting Duct Carcinoma"[All Fields]) OR ("Carcinoma, Collecting Duct"[All Fields]) OR ("Carcinomas, Collecting Duct"[All Fields]) OR ("Collecting Duct Carcinomas"[All Fields]) OR ("RCC"[All Fields]))) AND (("Anti-Bacterial Agents"[Mesh]) OR (("Anti-Bacterial Agents"[All Fields]) OR ("Agents, Anti-Bacterial"[All Fields]) OR ("Anti-Bacterial Agents"[All Fields]) OR ("Antibacterial Agents"[All Fields]) OR ("Agents, Antibacterial"[All Fields]) OR ("Antibacterial Agent"[All Fields]) OR ("Agent, Antibacterial"[All Fields]) OR ("Anti-Bacterial Compounds"[All Fields]) OR ("Anti-Bacterial Compounds"[All Fields]) OR ("Compounds, Anti-Bacterial"[All Fields]) OR ("Anti-Bacterial Agent"[All Fields]) OR ("Agent, Anti-Bacterial"[All Fields]) OR ("Anti-Bacterial Agent"[All Fields]) OR ("Anti-Bacterial Compound"[All Fields]) OR ("Anti-Bacterial Compound"[All Fields]) OR ("Compound, Anti-Bacterial"[All Fields]) OR ("Bacteriocidal Agents"[All Fields]) OR ("Agents, Bacteriocidal"[All Fields]) OR ("Bacteriocidal Agent"[All Fields]) OR ("Agent, Bacteriocidal"[All Fields]) OR ("Bacteriocide"[All Fields]) OR ("Bacteriocides"[All Fields]) OR ("Anti-Mycobacterial Agents"[All Fields]) OR ("Agents, Anti-Mycobacterial"[All Fields]) OR ("Anti Mycobacterial Agents"[All Fields]) OR ("Anti-Mycobacterial Agent"[All Fields]) OR ("Agent, Anti-Mycobacterial"[All Fields]) OR ("Anti Mycobacterial Agent"[All Fields]) OR ("Antimycobacterial Agent"[All Fields]) OR ("Agent, Antimycobacterial"[All Fields]) OR ("Antimycobacterial Agents"[All Fields]) OR ("Agents, Antimycobacterial"[All Fields]) OR ("Antibiotics"[All Fields]) OR ("Antibiotic"[All Fields]))))

Embase search strategy：

('renal cell carcinoma'/exp OR 'grawitz`s tumor' OR 'grawitz`s tumour' OR 'hyper-nephroma' OR 'hypernephroid cancer' OR 'kidney (renal cell) cancer' OR 'kidney cell adenocarcinoma' OR 'kidney cell cancer' OR 'kidney cell carcinoma' OR 'kidney hypernephroma' OR 'grawitz tumour' OR 'carcinoma, renal cell' OR 'carcinomas, renal cell' OR 'renal cell carcinomas' OR 'nephroid carcinoma' OR 'carcinoma, nephroid' OR 'nephroid carcinomas' OR 'adenocarcinoma of kidney' OR 'adenocarcinoma of kidneys' OR 'kidney, adenocarcinoma of' OR 'renal cell carcinoma' OR 'renal cell cancer' OR 'cancer, renal cell' OR 'renal cell cancers' OR 'adenocarcinoma, renal' OR 'renal adenocarcinoma' OR 'renal adenocarcinomas' OR 'renal carcinoma' OR 'carcinoma, renal' OR 'renal carcinomas' OR 'adenocarcinoma, renal cell' OR 'adenocarcinomas, renal cell' OR 'renal cell adenocarcinoma' OR 'renal cell adenocarcinomas' OR 'chromophobe renal cell carcinoma' OR 'sarcomatoid renal cell carcinoma' OR 'papillary renal cell carcinoma' OR 'renal cell carcinoma, papillary' OR 'chromophil renal cell carcinoma' OR 'clear cell renal cell carcinoma' OR 'grawitz tumor' OR 'tumor, grawitz' OR 'clear cell renal carcinoma' OR 'carcinoma, hypernephroid' OR 'hypernephroid carcinoma' OR 'hypernephroid carcinomas' OR 'hypernephroma' OR 'hypernephromas' OR 'collecting duct carcinoma (kidney)' OR 'carcinoma, collecting duct (kidney)' OR 'carcinomas, collecting duct (kidney)' OR 'collecting duct carcinomas (kidney)' OR 'collecting duct carcinoma of the kidney' OR 'renal collecting duct carcinoma' OR 'collecting duct carcinoma' OR 'carcinoma, collecting duct' OR 'carcinomas, collecting duct' OR 'collecting duct carcinomas' OR 'rcc') AND ('cancer

immunotherapy'/exp OR 'cancer immunotherapy' OR 'immunotherapy, cancer' OR 'tumor immunotherapy' OR 'tumour immunotherapy' OR 'immunotherapy' OR 'immunological therapy' OR 'immunotherapies' OR 'immune checkpoint inhibitors' OR 'checkpoint inhibitors, immune' OR 'immune checkpoint inhibitor' OR 'checkpoint inhibitor, immune' OR 'immune checkpoint blockers' OR 'checkpoint blockers, immune' OR 'immune checkpoint blockade' OR 'checkpoint blockade, immune' OR 'immune checkpoint inhibition' OR 'checkpoint inhibition, immune' OR 'pd-l1 inhibitors' OR 'pd l1 inhibitors' OR 'pd-l1 inhibitor' OR 'pd l1 inhibitor' OR 'programmed death-ligand 1 inhibitors' OR 'programmed death ligand 1 inhibitors' OR 'pd-1-pd-l1 blockade' OR 'blockade, pd-1-pd-l1' OR 'pd 1 pd l1 blockade' OR 'ctla-4 inhibitors' OR 'ctla 4 inhibitors' OR 'ctla-4 inhibitor' OR 'ctla 4 inhibitor' OR 'cytotoxic t-lymphocyte-associated protein 4 inhibitors' OR 'cytotoxic t lymphocyte associated protein 4 inhibitors' OR 'cytotoxic t-lymphocyte-associated protein 4 inhibitor' OR 'cytotoxic t lymphocyte associated protein 4 inhibitor' OR 'pd-1 inhibitors' OR 'pd 1 inhibitors' OR 'pd-1 inhibitor' OR 'inhibitor, pd-1' OR 'pd 1 inhibitor' OR 'programmed cell death protein 1 inhibitor' OR 'programmed cell death protein 1 inhibitors' OR 'abatacept' OR 'atezolizumab' OR 'ipilimumab' OR 'nivolumab' OR 'pembrolizumab' OR 'relatlimab' OR 'sotorasib' OR 'spartalizumab') AND ('antibiotic agent'/exp OR 'antibiotic agent' OR 'antibiotic combination' OR 'antibiotic drug' OR 'antibiotic ointment' OR 'antibiotic residue' OR 'antibiotic spectrum' OR 'antibiotics and their derivatives' OR 'antibiotics, combined' OR 'antibiotics, folate antagonists' OR 'antibiotics, miscellaneous' OR 'antibiotics, nitrofuran' OR 'antibiotics, oxalodinones' OR 'combined antibiotic' OR 'anti-bacterial agents' OR 'agents, anti-bacterial' OR 'anti-bacterial agents' OR 'antibacterial agents' OR 'agents, antibacterial' OR 'antibacterial agent' OR 'agent, antibacterial' OR 'anti-bacterial compounds' OR 'anti-bacterial compounds' OR 'compounds, anti-bacterial' OR 'anti-bacterial agent' OR 'agent, anti-bacterial' OR 'anti-bacterial agent' OR 'anti-bacterial compound' OR 'anti-bacterial compound' OR 'compound, anti-bacterial' OR 'bacteriocidal agents' OR 'agents, bacteriocidal' OR 'bacteriocidal agent' OR 'agent, bacteriocidal' OR 'bacteriocide' OR 'bacteriocides' OR 'anti-mycobacterial agents' OR 'agents, anti-mycobacterial' OR 'anti mycobacterial agents' OR 'anti-mycobacterial agent' OR 'agent, anti-mycobacterial' OR 'anti mycobacterial agent' OR 'antimycobacterial agent' OR 'agent, antimycobacterial' OR 'antimycobacterial agents' OR 'agents, antimycobacterial' OR 'antibiotics' OR 'antibiotic')

Web of Science search strategy：

TS=("Carcinoma, Renal Cell" OR "Carcinomas, Renal Cell" OR "Renal Cell Carcinomas" OR "Nephroid Carcinoma" OR "Carcinoma, Nephroid" OR "Nephroid Carcinomas" OR "Adenocarcinoma Of Kidney" OR "Adenocarcinoma Of Kidneys" OR "Kidney, Adenocarcinoma Of" OR "Renal Cell Carcinoma" OR "Renal Cell Cancer" OR "Cancer, Renal Cell" OR "Renal Cell Cancers" OR "Adenocarcinoma, Renal" OR "Renal Adenocarcinoma" OR "Renal Adenocarcinomas" OR "Renal Carcinoma" OR "Carcinoma, Renal" OR "Renal Carcinomas" OR "Adenocarcinoma, Renal Cell" OR "Adenocarcinomas, Renal Cell" OR "Renal Cell Adenocarcinomas" OR "Renal Cell Adenocarcinoma" OR "Chromophobe Renal Cell Carcinoma" OR "Sarcomatoid Renal Cell Carcinoma" OR "Papillary Renal Cell Carcinoma" OR "Renal Cell Carcinoma, Papillary" OR "Chromophil Renal Cell Carcinoma" OR "Clear Cell Renal Cell Carcinoma" OR "Grawitz Tumor" OR "Tumor, Grawitz" OR "Clear Cell Renal Carcinoma" OR "Hypernephroid Carcinoma" OR "Carcinoma, Hypernephroid" OR "Hypernephroid Carcinomas" OR "Hypernephroma" OR "Hypernephromas" OR "Collecting Duct Carcinoma (Kidney)" OR "Carcinoma, Collecting Duct (Kidney)" OR "Carcinomas, Collecting Duct (Kidney)" OR "Collecting Duct Carcinomas (Kidney)" OR "Collecting Duct Carcinoma of the Kidney" OR "Collecting Duct Carcinoma" OR "Renal Collecting Duct Carcinoma" OR "Carcinoma, Collecting Duct" OR "Carcinomas, Collecting Duct" OR "Collecting Duct Carcinomas" OR "RCC") AND TS=("Anti-Bacterial Agents" OR "Agents, Anti-Bacterial" OR "Anti-Bacterial Agents" OR "Antibacterial Agents" OR "Agents, Antibacterial" OR "Antibacterial Agent" OR "Agent, Antibacterial" OR "Anti-Bacterial Compounds" OR "Anti-Bacterial Compounds" OR "Compounds, Anti-Bacterial" OR "Anti-Bacterial Agent" OR "Agent, Anti-Bacterial" OR "Anti-Bacterial Agent" OR "Anti-Bacterial Compound" OR "Anti-Bacterial Compound" OR "Compound, Anti-Bacterial" OR "Bacteriocidal Agents" OR "Agents, Bacteriocidal" OR "Bacteriocidal Agent" OR "Agent, Bacteriocidal" OR "Bacteriocide" OR "Anti-Mycobacterial Agents" OR "Bacteriocides" OR "Agents, Anti-Mycobacterial" OR "Anti Mycobacterial Agents" OR "Anti-Mycobacterial Agent" OR "Agent, Anti-Mycobacterial" OR "Anti Mycobacterial Agent" OR "Antimycobacterial Agent" OR "Agent, Antimycobacterial" OR "Antimycobacterial Agents" OR "Agents, Antimycobacterial" OR "Antibiotic" OR "Antibiotics") AND TS=("Immunotherapy" OR "immunological therapy" OR "immunotherapies" OR "Immune Checkpoint Inhibitors" OR "Checkpoint Inhibitors, Immune" OR "Immune Checkpoint Inhibitor" OR

"Checkpoint Inhibitor, Immune" OR "Immune Checkpoint Blockers" OR "Checkpoint Blockers, Immune" OR "Immune Checkpoint Blockade" OR "Checkpoint Blockade, Immune" OR "Immune Checkpoint Inhibition" OR "Checkpoint Inhibition, Immune" OR "PD-L1 Inhibitors" OR "PD L1 Inhibitors" OR "PD-L1 Inhibitor" OR "PD L1 Inhibitor" OR "Programmed Death-Ligand 1 Inhibitors" OR "Programmed Death Ligand 1 Inhibitors" OR "PD-1-PD-L1 Blockade" OR "Blockade, PD-1-PD-L1" OR "CTLA-4 Inhibitors" OR "PD 1 PD L1 Blockade" OR "CTLA 4 Inhibitors" OR "CTLA-4 Inhibitor" OR "CTLA 4 Inhibitor" OR "Cytotoxic T-Lymphocyte-Associated Protein 4 Inhibitors" OR "Cytotoxic T Lymphocyte Associated Protein 4 Inhibitors" OR "Cytotoxic T-Lymphocyte-Associated Protein 4 Inhibitor" OR "Cytotoxic T Lymphocyte Associated Protein 4 Inhibitor" OR "PD-1 Inhibitors" OR "PD 1 Inhibitors" OR "Inhibitor, PD-1" OR "PD-1 Inhibitor" OR "PD 1 Inhibitor" OR "Programmed Cell Death Protein 1 Inhibitor" OR "Programmed Cell Death Protein 1 Inhibitors" OR "Abatacept" OR "atezolizumab" OR "Ipilimumab" OR "Nivolumab" OR "pembrolizumab" OR "sotorasib" OR "relatlimab " OR "spartalizumab")

Scopus search strategy:

(TITLE-ABS-KEY ("Immunotherapy" OR "immunological therapy" OR "immunotherapies" OR "Immune Checkpoint Inhibitors" OR "Checkpoint Inhibitors, Immune" OR "Immune Checkpoint Inhibitor" OR "Checkpoint Inhibitor, Immune" OR "Immune Checkpoint Blockers" OR "Checkpoint Blockers, Immune" OR "Immune Checkpoint Blockade" OR "Checkpoint Blockade, Immune" OR "Immune Checkpoint Inhibition" OR "Checkpoint Inhibition, Immune" OR "PD-L1 Inhibitors" OR "PD L1 Inhibitors" OR "PD-L1 Inhibitor" OR "PD L1 Inhibitor" OR "Programmed Death-Ligand 1 Inhibitors" OR "Programmed Death Ligand 1 Inhibitors" OR "PD-1-PD-L1 Blockade" OR "Blockade, PD-1-PD-L1" OR "PD 1 PD L1 Blockade" OR "CTLA-4 Inhibitors" OR "CTLA 4 Inhibitors" OR "CTLA-4 Inhibitor" OR "CTLA 4 Inhibitor" OR "Cytotoxic T-Lymphocyte-Associated Protein 4 Inhibitors" OR "Cytotoxic T Lymphocyte Associated Protein 4 Inhibitors" OR "Cytotoxic T-Lymphocyte-Associated Protein 4 Inhibitor" OR "Cytotoxic T Lymphocyte Associated Protein 4 Inhibitor" OR "PD-1 Inhibitors" OR "PD 1 Inhibitors" OR "PD-1 Inhibitor" OR "Inhibitor, PD-1" OR "PD 1 Inhibitor" OR "Programmed Cell Death Protein 1 Inhibitor" OR "Programmed Cell Death Protein 1 Inhibitors" OR "Abatacept" OR "atezolizumab" OR "Ipilimumab" OR "Nivolumab" OR "pembrolizumab" OR "relatlimab " OR "sotorasib" OR "spartalizumab ")) AND (TITLE-ABS-KEY ("Carcinoma, Renal Cell" OR "Carcinomas, Renal Cell" OR "Renal Cell Carcinomas" OR "Nephroid Carcinoma" OR "Carcinoma, Nephroid" OR "Nephroid Carcinomas" OR "Adenocarcinoma Of Kidney" OR "Adenocarcinoma Of Kidneys" OR "Kidney, Adenocarcinoma Of" OR "Renal Cell Carcinoma" OR "Renal Cell Cancer" OR "Cancer, Renal Cell" OR "Renal Cell Cancers" OR "Adenocarcinoma, Renal" OR "Renal Adenocarcinoma" OR "Renal Adenocarcinomas" OR "Renal Carcinoma" OR "Carcinoma, Renal" OR "Renal Carcinomas" OR "Adenocarcinoma, Renal Cell" OR "Adenocarcinomas, Renal Cell" OR "Renal Cell Adenocarcinoma" OR "Renal Cell Adenocarcinomas" OR "Chromophobe Renal Cell Carcinoma" OR "Sarcomatoid Renal Cell Carcinoma" OR "Papillary Renal Cell Carcinoma" OR "Renal Cell Carcinoma, Papillary" OR "Chromophil Renal Cell Carcinoma" OR "Clear Cell Renal Cell Carcinoma" OR "Grawitz Tumor" OR "Tumor, Grawitz" OR "Clear Cell Renal Carcinoma" OR "Carcinoma, Hypernephroid" OR "Hypernephroid Carcinoma" OR "Hypernephroid Carcinomas" OR "Hypernephroma" OR "Hypernephromas" OR "Collecting Duct Carcinoma (Kidney)" OR "Carcinoma, Collecting Duct (Kidney)" OR "Carcinomas, Collecting Duct (Kidney)" OR "Collecting Duct Carcinomas (Kidney)" OR "Collecting Duct Carcinoma of the Kidney" OR "Renal Collecting Duct Carcinoma" OR "Collecting Duct Carcinoma" OR "Carcinoma, Collecting Duct" OR "Carcinomas, Collecting Duct" OR "Collecting Duct Carcinomas" OR "RCC")) AND (TITLE-ABS-KEY ("Anti-Bacterial Agents" OR "Agents, Anti-Bacterial" OR "Anti Bacterial Agents" OR "Antibacterial Agents" OR "Agents, Antibacterial" OR "Antibacterial Agent" OR "Agent, Antibacterial" OR "Anti-Bacterial Compounds" OR "Anti-Bacterial Compounds" OR "Compounds, Anti-Bacterial" OR "Anti-Bacterial Agent" OR "Agent, Anti-Bacterial" OR "Anti-Bacterial Agent" OR "Anti-Bacterial Compound" OR "Anti-Bacterial Compound" OR "Compound, Anti-Bacterial" OR "Bacteriocidal Agents" OR "Agents, Bacteriocidal" OR "Bacteriocidal Agent" OR "Agent, Bacteriocidal" OR "Bacteriocide" OR "Bacteriocides" OR "Anti-Mycobacterial Agents" OR "Agents, Anti-Mycobacterial" OR "Anti Mycobacterial Agents" OR "Anti-Mycobacterial Agent" OR "Agent, Anti-Mycobacterial" OR "Anti Mycobacterial Agent" OR "Antimycobacterial Agent" OR "Agent, Antimycobacterial" OR "Antimycobacterial Agents" OR "Agents, Antimycobacterial" OR "Antibiotics" OR "Antibiotic"))

Cochrane Library search strategy：

#1 MeSH descriptor: [Immune Checkpoint Inhibitors] explode all trees

#2 ("Immunotherapy" OR "immunological therapy" OR "immunotherapies" OR "Immune Checkpoint Inhibitors" OR "Checkpoint Inhibitors, Immune" OR "Immune Checkpoint Inhibitor" OR "Checkpoint Inhibitor, Immune" OR "Immune Checkpoint Blockers" OR "Checkpoint Blockers, Immune" OR "Immune Checkpoint Blockade" OR "Checkpoint Blockade, Immune" OR "Immune Checkpoint Inhibition" OR "Checkpoint Inhibition, Immune" OR "PD-L1 Inhibitors" OR "PD L1 Inhibitors" OR "PD-L1 Inhibitor" OR "PD L1 Inhibitor" OR "Programmed Death-Ligand 1 Inhibitors" OR "Programmed Death Ligand 1 Inhibitors" OR "PD-1-PD-L1 Blockade" OR "Blockade, PD-1-PD-L1" OR "CTLA-4 Inhibitors" OR "PD 1 PD L1 Blockade" OR "CTLA 4 Inhibitors" OR "CTLA-4 Inhibitor" OR "CTLA 4 Inhibitor" OR "Cytotoxic T-Lymphocyte-Associated Protein 4 Inhibitors" OR "Cytotoxic T Lymphocyte Associated Protein 4 Inhibitors" OR "Cytotoxic T-Lymphocyte-Associated Protein 4 Inhibitor" OR "Cytotoxic T Lymphocyte Associated Protein 4 Inhibitor" OR "PD-1 Inhibitors" OR "PD 1 Inhibitors" OR "Inhibitor, PD-1" OR "PD-1 Inhibitor" OR "PD 1 Inhibitor" OR "Programmed Cell Death Protein 1 Inhibitor" OR "Programmed Cell Death Protein 1 Inhibitors" OR "Abatacept" OR "atezolizumab" OR "Ipilimumab" OR "Nivolumab" OR "pembrolizumab" OR "sotorasib" OR "relatlimab " OR "spartalizumab"):ti,ab,kw

#3 #1 or #2

#4 MeSH descriptor: [Anti-Bacterial Agents] explode all trees

#5 ("Anti-Bacterial Agents" OR "Agents, Anti-Bacterial" OR "Anti-Bacterial Agents" OR "Antibacterial Agents" OR "Agents, Antibacterial" OR "Antibacterial Agent" OR "Agent, Antibacterial" OR "Anti-Bacterial Compounds" OR "Anti-Bacterial Compounds" OR "Compounds, Anti-Bacterial" OR "Anti-Bacterial Agent" OR "Agent, Anti-Bacterial" OR "Anti-Bacterial Agent" OR "Anti-Bacterial Compound" OR "Anti-Bacterial Compound" OR "Compound, Anti-Bacterial" OR "Bacteriocidal Agents" OR "Agents, Bacteriocidal" OR "Bacteriocidal Agent" OR "Agent, Bacteriocidal" OR "Bacteriocide" OR "Anti-Mycobacterial Agents" OR "Bacteriocides" OR "Agents, Anti-Mycobacterial" OR "Anti Mycobacterial Agents" OR "Anti-Mycobacterial Agent" OR "Agent, Anti-Mycobacterial" OR "Anti Mycobacterial Agent" OR "Antimycobacterial Agent" OR "Agent, Antimycobacterial" OR "Antimycobacterial Agents" OR "Agents, Antimycobacterial" OR "Antibiotic" OR "Antibiotics"):ti,ab,kw

#6 #4 or #5

#7 MeSH descriptor: [Carcinoma, Renal Cell] explode all trees

#8 ("Carcinoma, Renal Cell" OR "Carcinomas, Renal Cell" OR "Renal Cell Carcinomas" OR "Nephroid Carcinoma" OR "Carcinoma, Nephroid" OR "Nephroid Carcinomas" OR "Adenocarcinoma Of Kidney" OR "Adenocarcinoma Of Kidneys" OR "Kidney, Adenocarcinoma Of" OR "Renal Cell Carcinoma" OR "Renal Cell Cancer" OR "Cancer, Renal Cell" OR "Renal Cell Cancers" OR "Adenocarcinoma, Renal" OR "Renal Adenocarcinoma" OR "Renal Adenocarcinomas" OR "Renal Carcinoma" OR "Carcinoma, Renal" OR "Renal Carcinomas" OR "Adenocarcinoma, Renal Cell" OR "Adenocarcinomas, Renal Cell" OR "Renal Cell Adenocarcinomas" OR "Renal Cell Adenocarcinoma" OR "Chromophobe Renal Cell Carcinoma" OR "Sarcomatoid Renal Cell Carcinoma" OR "Papillary Renal Cell Carcinoma" OR "Renal Cell Carcinoma, Papillary" OR "Chromophil Renal Cell Carcinoma" OR "Clear Cell Renal Cell Carcinoma" OR "Grawitz Tumor" OR "Tumor, Grawitz" OR "Clear Cell Renal Carcinoma" OR "Hypernephroid Carcinoma" OR "Carcinoma, Hypernephroid" OR "Hypernephroid Carcinomas" OR "Hypernephroma" OR "Hypernephromas" OR "Collecting Duct Carcinoma (Kidney)" OR "Carcinoma, Collecting Duct (Kidney)" OR "Carcinomas, Collecting Duct (Kidney)" OR "Collecting Duct Carcinomas (Kidney)" OR "Collecting Duct Carcinoma of the Kidney" OR "Collecting Duct Carcinoma" OR "Renal Collecting Duct Carcinoma" OR "Carcinoma, Collecting Duct" OR "Carcinomas, Collecting Duct" OR "Collecting Duct Carcinomas" OR "RCC"):ti,ab,kw

#9 #7 or #8

#10 #3 and #6 and #9

Supplementary table 1a. Detailed information of basic characteristics of included studies.

Basic information				Population					Characteristics of immunotherapy	
**Author, year**	**Type of study**	**Type of publication**	**Area**	**Number of patients**	**Gender<br>Age: median(range)**	**IMDC risk**	**Cancer histology**	**Data on metastasis**	**Type of immunotherapy**	**Line of treatment**
UEDA et al, 2019	retrospective	publication	Japan	n=31(in total)<br>n=5(29.9% with ATB)	male=77.4%<br>female=22.6%<br>Age: 67(44-80) yr	mRCC:<br>favorable: 9.7%<br>intermediate: 64.5%<br>poor: 25.8%	87.1% clear cell<br>6.5% Papillary<br>6.5%unknown	NA	Nivolumab: 90.3%<br>Ipilimumab + nivolumab: 9.7%	first-line:9.7%<br>second- line:90.3%
Lalani et al, 2020	retrospective	publication	the USA	n=146(in total)<br>n=31(21% with ATB)	male=71.2%<br>female=28.8%<br>Age: 61(22-82) yr	mRCC:<br>favorable: 17.8%<br>intermediate: 58.2%<br>poor: 21.2%<br>unknown: 2.7%	84.9% clear cell<br>15.1% no-clear cell with sarcomatoid differentiation	NA	anti-PD-(L)1–monotherapy:54.8 %<br>anti-PD-(L)1–combination:44.2%	first-line:43.2%<br>second- line:26.7%<br>third-line or later:30.1%
				n=146(in total)<br>n=27(18.5% with ATB)	male=71.2%<br>female=28.8%<br>Age: 61(22-82) yr	mRCC:<br>favorable: 17.8%<br>intermediate: 58.2%<br>poor: 21.2%<br>unknown: 2.7%	84.9% clear cell<br>15.1% no-clear cell with sarcomatoid differentiation	NA	anti-PD-(L)1–monotherapy:54.8 %<br>anti-PD-(L)1–combination:44.2%	first-line:43.2%<br>second- line:26.7%<br>third-line or later:30.1%
Kulkarni et al,<br>2020	retrospective	publication	the USA	n=55(in total)<br>n=24(44% with ATB)	male=73%<br>female=27%<br>Age：62(23–86) yr	advanced or metastatic	96% clear cell<br>4% Papillary	NA	nivolumab: 93%<br>others: 7%	first-line:7%<br>second-line or later:93%
Guven et al, 2021	retrospective	publication	Turkey	n=93(in total)<br>n=31(33.3% with ATB)	male=76%<br>female=24%<br>Age:<br>ABT+:61 (28-81)<br>ABT-:63 (32-77)	mRCC:<br>favorable: 22.6%<br>poor and intermediate: 77.4%	NA	Lung:81.7%<br>Liver:28.0%<br>Brain:13%	nivolumab and others	second- line:54.8%<br>third-line or later:45.2%
Derosa et al, 2021	retrospective	abstract	France	n=707(in total)<br>n=104(14.7% with ATB)	NA	mRCC:<br>favorable: 18%<br>intermediate: 56%<br>poor: 26%	NA	NA	nivolumab	NA
Taigo Kato et al,<br>2022	retrospective	publication	Japan	n=72(in total)<br>n=47(65.3% with ATB)	male=84.7%<br>female=15.3%<br>Age: 70(36–86) yr	mRCC:<br>intermediate: 69.4%<br>poor: 30.6%	76.4% clear cell<br>20.8% no-clear cell<br>2.8% sarcomatoid component	Lung:65.3%<br>Liver:13.8%<br>Bone:25%<br>Brain:4.2%<br>Lymph node:36%<br>Adrenal:5.6%	nivolumab plus ipilimumab:100%	first-line:100%
Derosa et al, 2018	retrospective	publication	France	n=121(in total)<br>n=16(13% with ATB)	male=66%<br>female=34%<br>Age: 61(28-83) yr	mRCC:<br>favorable: 21%<br>intermediate: 59%<br>poor: 20%	95% clear cell<br>5% no-clear cell	Lung:69%<br>Liver:28%<br>Bone:27%<br>Brain:13%	anti-PD-(L)1 therapy: 88%<br>anti-PD-(L)1+CTLA-4: 8%<br>anti-PD-(L)1 + bevacizumab: 4%	first-line:57%<br>second-line or later:43%
				n=121(in total)<br>n=22(18% with ATB)	male=66%<br>female=34%<br>Age: 61(28-83) yr	mRCC:<br>favorable: 21%<br>intermediate: 59%<br>poor: 20%	95% clear cell<br>5% no-clear cell	Lung:69%<br>Liver:28%<br>Bone:27%<br>Brain:13%	anti-PD-(L)1 therapy: 88%<br>anti-PD-(L)1+CTLA-4: 8%<br>anti-PD-(L)1 + bevacizumab: 4%	first-line:57%<br>second-line or later:43%
Routy et al, 2018	retrospective	publication	multicenter	n=67(in total)<br>n=20(29.9% with ATB)	male=67%<br>female=33%<br>Age: 61(29-83) yr	mRCC:<br>favorable: 21%<br>intermediate: 58%<br>poor: 21%	91% clear cell<br>9% Papillary	Lung:69%<br>Liver:30%<br>Bone:31%<br>Brain:11%	anti-PD-1: 92%<br>anti-PD-L1: 8%	NA
Kulkarni et al,<br>2019	retrospective	abstract	the USA	n=55(in total)<br>n=40(72% with ATB)	NA	NA	NA	NA	anti-PD-(L)1	NA
Derosa et al, 2020	retrospective	publication	France	n=69(in total)<br>n=11(16% with ATB)	male=69.6%<br>female=30.4%<br>Age: 62(30-82) yr	stage IV RCC:<br>favorable: 20.3%<br>intermediate: 56.5%<br>poor: 20.3%<br>unknown: 2.9%	97.1% clear cell<br>2.9% no-clear cell	Lung:74%<br>Liver:19%<br>Bone:28%<br>lymph node:52%	nivolumab	NA

ATBs, antibiotics; yr, year; IMDC risk, International Metastatic RCC Database Consortium risk; mRCC, metastatic renal cell carcinoma; PD-1, programmed death 1; PD-L1, programmed cell death-Ligand 1; CTLA-4, cytotoxic T lymphocyte-associated antigen-4; NA, not available.

Supplementary table 1b. Detailed information of basic characteristics of included studies.

	Characteristics of ABT use				Outcomes							
**Author, year**	**Time window of ABT exposure to ICI initiation**	**Reason for ABT use**	**Type of ATB**	**Duration of ATB**	**mFollow-up (range) (months)**	**mPFS ABT+ vs. ABT-Δ(months)**	**mOS ABT+ vs. ABT-Δ(months)**	**HR for PFS (95% CI)**	**HR for OS (95% CI)**	**HR Source**	**ORR ABT+ vs. ABT-**	**PD ABT+ vs. ABT-**
UEDA et al, 2019	-30d to 0	A	β-Lactam inhibitors (100%)	NA	NA	2.8 vs.18.4<br>Δ=15.6	NA	(Univariate)<br>6.518(1.857-21.416)<br>(Multivariate)<br>3.830<br>(1.086-12.717)	not affected	Available	not affected.	NA
Lalani et al, 2020	-8w to +4w	NA	β-lactam ± inhibitors (33.5%)<br>fluoroquinolones (22.6%)<br>macrolides (9.7%)<br>tetracyclines (9.7%)<br>other and unknow (22.6%)	NA	16.6<br>(0.7–67.8)	2.6vs.8.1<br>Δ=4.5	NA	(Multivariate)<br>1.96(1.20–3.20)	(Multivariate)<br>1.44 (0.75–2.77)	Available	12.9% vs.34.8%<br>p=0.026	NA
	-30d to +30d	NA	β-lactam ± inhibitors (33.5%)<br>fluoroquinolones (22.6%)<br>macrolides (9.7%)<br>tetracyclines (9.7%)<br>other and unknow (22.6%)	NA	16.6<br>(0.7–67.8)	NA	NA	(Multivariate)<br>2.03 (1.21–3.41)	(Multivariate)<br>1.59 (0.80–3.15)	Available	NA	NA
Kulkarni et al, 2020	-1m to +6w	NA	β-lactam ± inhibitors (96%)<br>Quinolones (36%)<br>Tetracyclines (4%)<br>Vancomycin (4%)<br>Macrolides (4%)<br>Sulfonamides (4%)<br>Metronidazole (12%)	NA	18.7	2.7 vs 4.2<br>Δ=1.5	17 vs 22<br>Δ=5	(Multivariate)<br>2.7(1.3-5.9)	(Multivariate)<br>4.2(1.5-12.2)	Available	NA	NA
Guven et al, 2021	-3m to +3m	NA	Quinolones (48.4%)<br>β-lactam ± inhibitors(45.1%)<br>clarithromycin(3.2%)<br>metronidazole(3.2%)	NA	10.87	NA	NA	(Multivariate)<br>2.238 (1.284-3.900)	(Multivariate)<br>2.306<br>(1.155-4.601)	Available	24.1% vs.50%,<br>P=0.023	41.4%<br>vs.23.1%<br>P=0.084
Derosa et al, 2021	-60d to +42d	NA	NA	NA	NA	2.6 vs.3.8<br>Δ=1.2	13.0 vs.25.0<br>Δ=12	(Univariate)<br>1.24 (0.99-1.55)	(Univariate)<br>1.77 (1.36-2.31)<br>(Multivariate)<br>1.59 (1.22-2.09)	Available	15.1vs.21.1%,<br>P=0.176	57% vs.<br>47.3%
Taigo Kato et al,2022	-3m to 0	E	beta-lactams:95.7%<br>others:4.3%	NA	16.1<br>(1.4–37.8)	13.2 vs. NR	NA	(Univariate)<br>0.86(0.29-2.53)	(Univariate)<br>0.66(0.13-3.35)	Estimated	NA	NA
Derosa et al, 2018	-30d to 0	A: 38%<br>B: 19%<br>C: 31%<br>D: 12%	β-lactam ± inhibitors (82%)<br>Quinolones (6%)<br>Tetracyclines (6%)<br>Aminoglycosides (6%)	≤ 7d (50%)<br>> 7d (50%)	NA	1.9 vs 7.4<br>Δ=5.5	17.3 vs.30.6<br>Δ=13.3	(Univariate)<br>3.1 (1.4-6.9)<br>(Multivariate)<br>2.2 (1.3–3.3)	(Univariate)<br>3.5 (1.1-10.8)<br>(Multivariate)<br>2.1 (0.9–5.0)	Available	13% vs.26%	75%<br>vs.22%<br>P < 0.01
	-60d to 0	A: 27%<br>B: 23%<br>C: 9%<br>D: 18%	β-lactam ± inhibitors (88%)<br>Quinolones (4%)<br>Tetracyclines (4%)<br>Aminoglycosides (4%)	≤ 7d (50%)<br>> 7d (46%)<br>not reported (4%)	NA	3.1 vs 7.4<br>Δ=4.3	23.4 vs.30.0<br>Δ=6.6	(Univariate)<br>2.3 (1.2-4.4)<br>(Multivariate)<br>3.2 (1.6-5.9)	(Univariate)<br>2.0 (0.9-4.3)	Available	18% vs.25%	64%<br>vs.21%<br>P < 0.01
Routy et al, 2018	-2m to +1m	A	β-lactam ± inhibitors (60%)<br>Quinolones (10%)<br>Tetracyclines (5%)<br>Aminoglycosides (5%)<br>Nitrofurans (5%)<br>others (15%)	≤ 7d (45%)<br>> 7d (30%)<br>not reported (25%)	NA	4.3 vs 7.4<br>Δ=3.1	23.4 vs 27.9<br>Δ=4.5	(Univariate)<br>2.16 (1.18-3.96)<br>(Multivariate)<br>2.12 (1.11–4.05)	(Univariate)<br>1.22(0.84–1.91)	Available for PFS, Estimated for OS	NA	NA
Kulkarni et al, 2019	-< 1m to during	NA	NA	NA	NA	2.9 v 5.0<br>Δ=2.1	not affected.	(Univariate)<br>2.3(1.0-5.0)	not affected	Available	not affected.	NA
Derosa et al, 2020	NA	A: 46%<br>B: 9%<br>C: 18%<br>D: 18%<br>F: 9%	β-lactam ± inhibitors (82%)<br>fluoroquinolones (9%)<br>unknown (9%)	≤ 7d (36%)<br>> 7d (64%)	23.54<br>(0.66-32.21)	1.87 vs 5.09<br>Δ=3.22	24.6 vs<br>undefined	(Univariate)<br>3.85(1.69–8.78)	(Univariate)<br>3.84<br>(1.16–12.70)	Estimated	9% vs.28%<br>P< 0.03	73%<br>vs.33%

A, infection; B, TKI or EGFR associated skin toxicity; C, Flu-like syndrome. D, m-TOR inhibitor associated; E, perioperative use; F, unknow; ATB, antibiotics; ATB+, antibiotics present; ATB-, antibiotics absent; w, week(s); m, month(s); d, days; NA, not available; NR, not reached; mFollow-up, median Follow-up; mPFS, median progression free survival; mOS, median overall survival; OS, overall survival; PFS, progression-free survival; HR, hazard ratio; CI, confidence interval; ORR, objective response rate; PD, primary progressive disease; P, p-Value.

Supplementary table 2：Study quality of included studies based on the Newcastle-Ottawa scale

Author, year	Representativeness of the exposed cohort	Selection of the nonexposed cohort	Ascertainment of exposure	Demonstration that outcome of interest was not present at start of study	Comparability of cohorts on the basis of the design or analysis	Assessment of outcome	Was follow-up long enough for outcomes to occur	Adequacy of follow-up of cohorts	Total score
Lalani et al, 2020	★	★	★	★	★ ★	★	★	★	9
Derosa et al, 2020	★	★	★	★	☆ ☆	★	★	☆	6
Derosa et al, 2018	★	★	★	★	★ ★	★	★	☆	8
Derosa et al, 2021	★	★	★	★	★ ★	★	☆	☆	7
Guven et al, 2021	★	★	★	★	★ ★	★	★	☆	8
Kulkarni et al, 2019	★	★	★	★	★ ★	★	☆	☆	7
Kulkarni et al, 2020	★	★	★	★	★ ★	★	★	☆	8
Routy et al, 2018	★	★	★	☆	★ ★	★	★	★	8
UEDA et al, 2019	★	★	★	★	★ ★	★	☆	☆	7
Taigo Kato et al, 2022	★	★	★	★	☆ ☆	★	☆	☆	5

Supplementary figure 1.

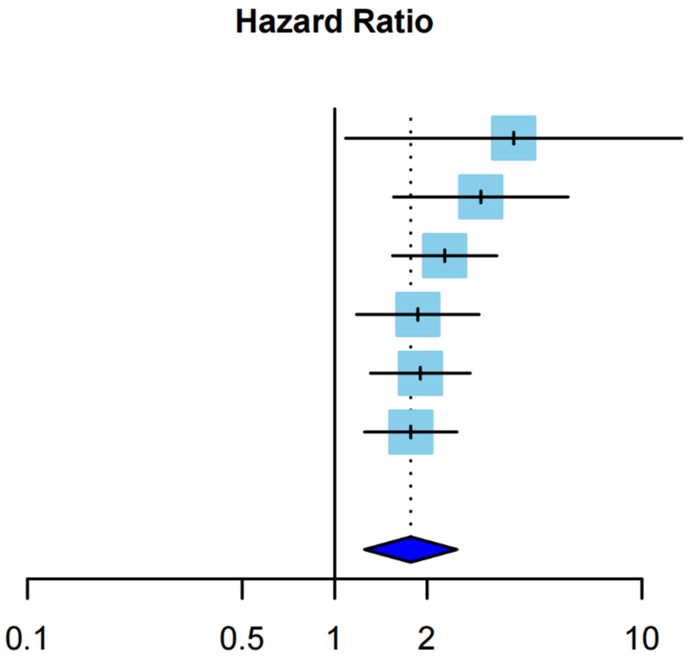

Study	HR	95%−CI	P−value	Tau2	Tau	I2
Adding UEDA et al,2019 (k=1)	3.83	[1.09; 13.48]	0.04	.	.	.
Adding Kulkarni et al,2020 (k=2)	2.99	[1.55; 5.75]	< 0.01	0	0	0%
Adding Lalani et al,2020 (k=3)	2.28	[1.54; 3.37]	< 0.01	0	0	0%
Adding Derosa et al,2021 (k=4)	1.86	[1.18; 2.95]	< 0.01	0.1235	0.3515	64%
Adding Guven et al,2021 (k=5)	1.90	[1.31; 2.76]	< 0.01	0.0941	0.3067	61%
Adding Taigo Kato et al,2022 (k=6)	1.77	[1.25; 2.50]	< 0.01	0.0841	0.2900	56%
**Random effects model**	**1.77**	**[1.25; 2.50]**	**< 0.01**	**0.0841**	**0.2900**	**56%**

Supplementary figure 2.

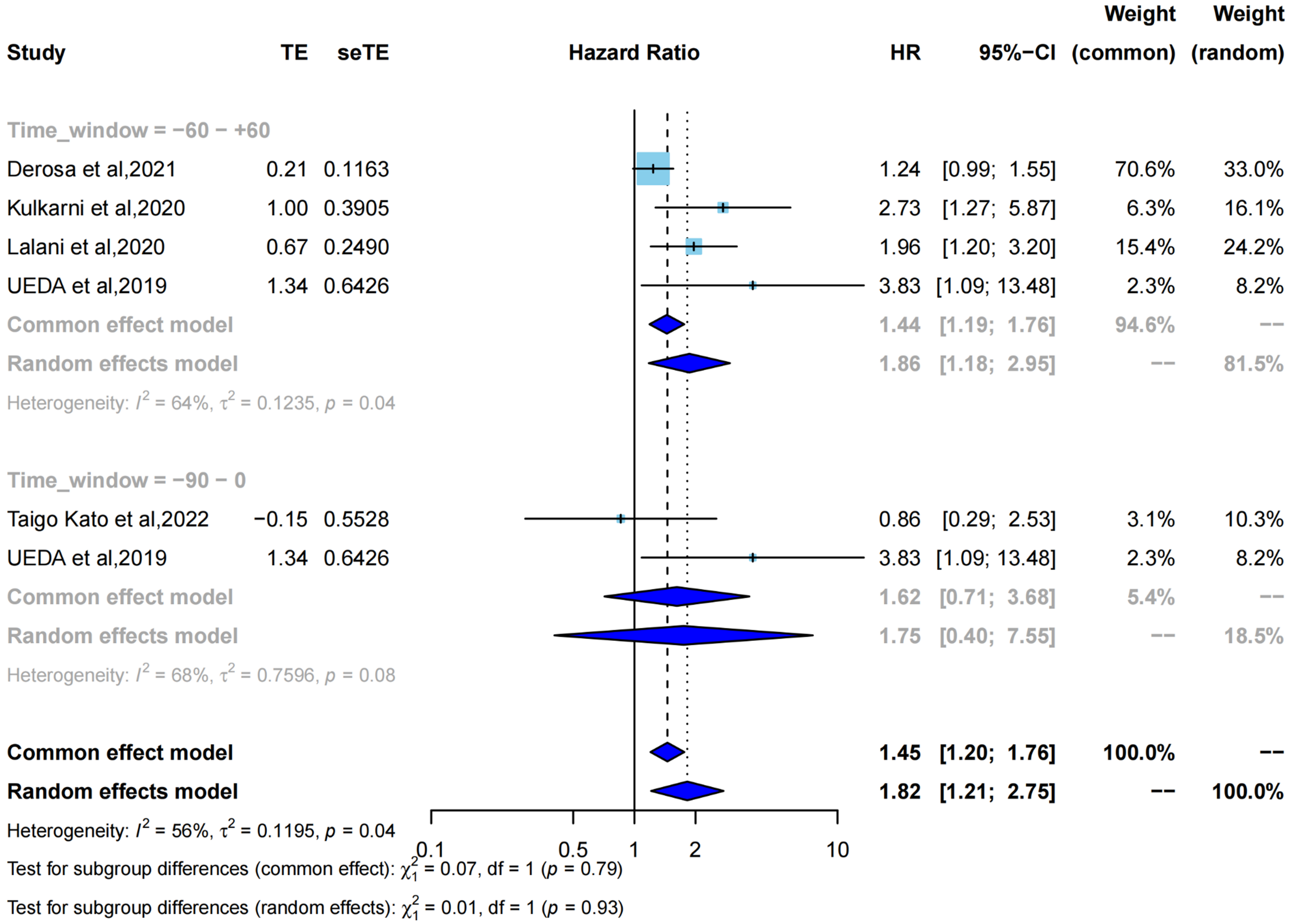

Supplementary figure 3.

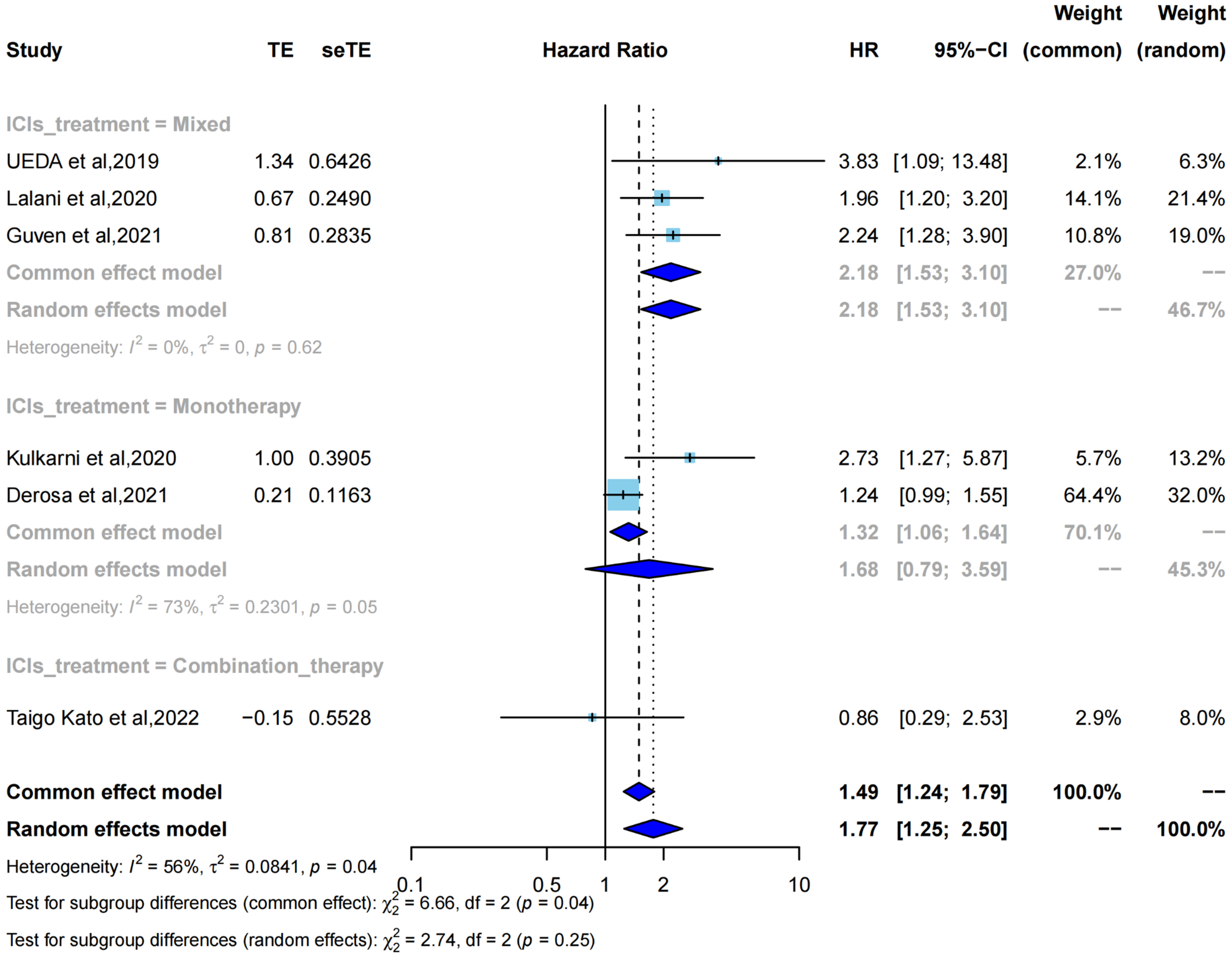

Supplementary figure 4

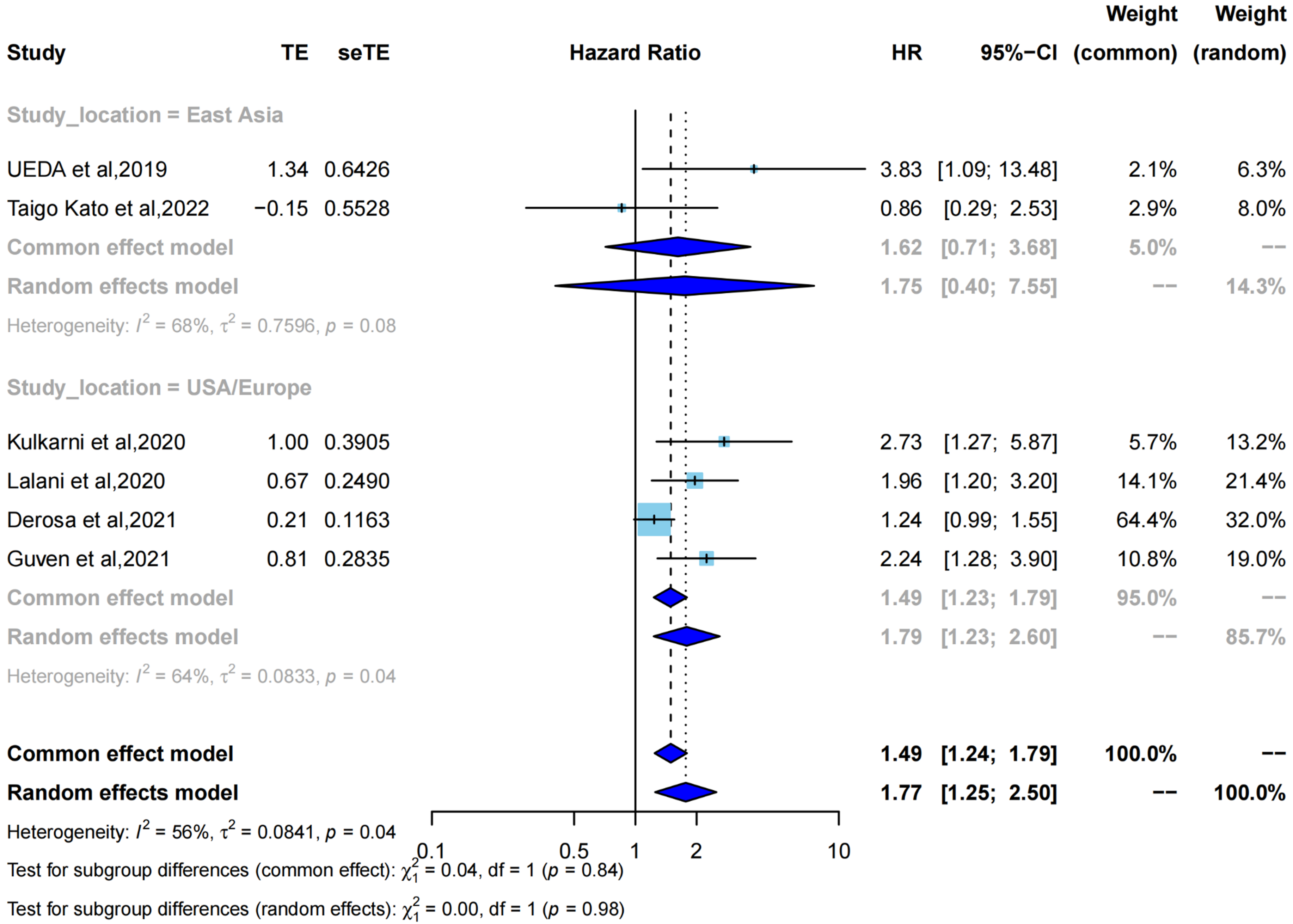

Supplementary figure 5.

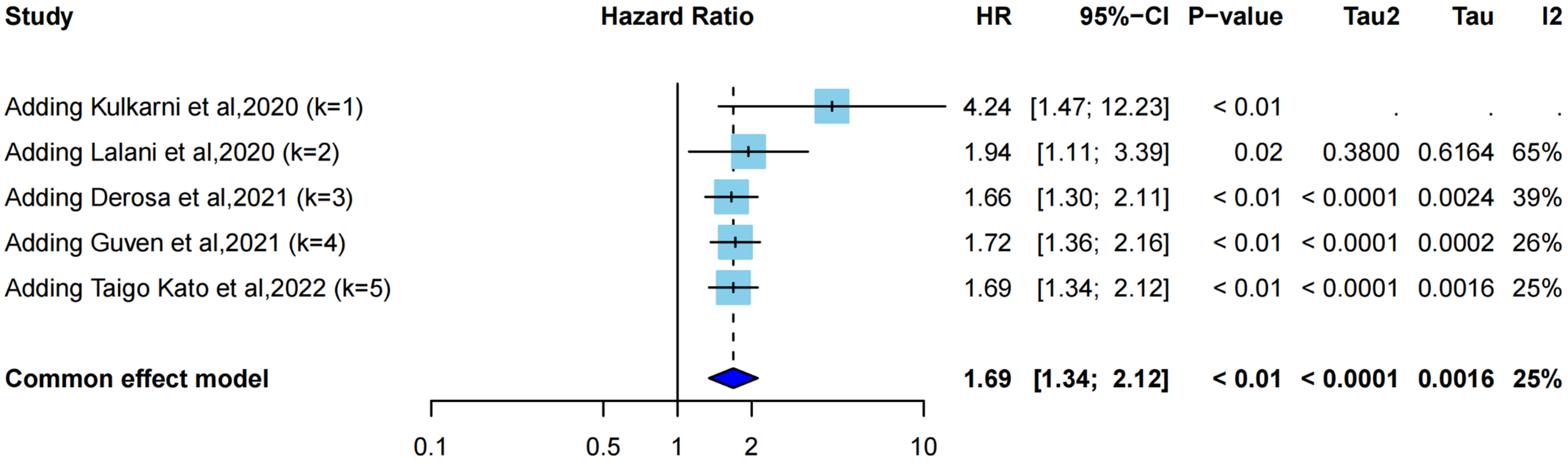

Supplementary figure 6.

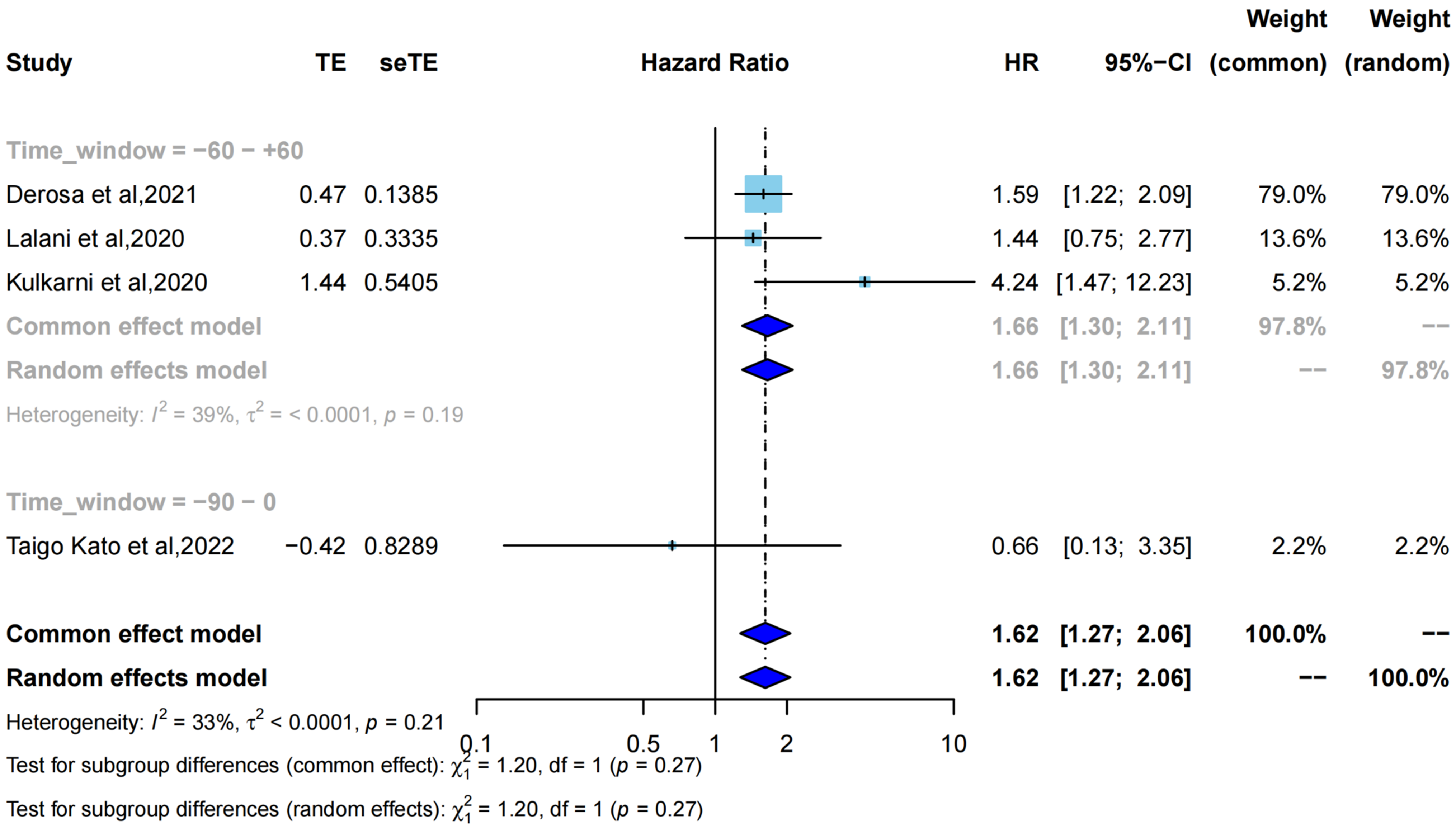

Supplementary figure 7.

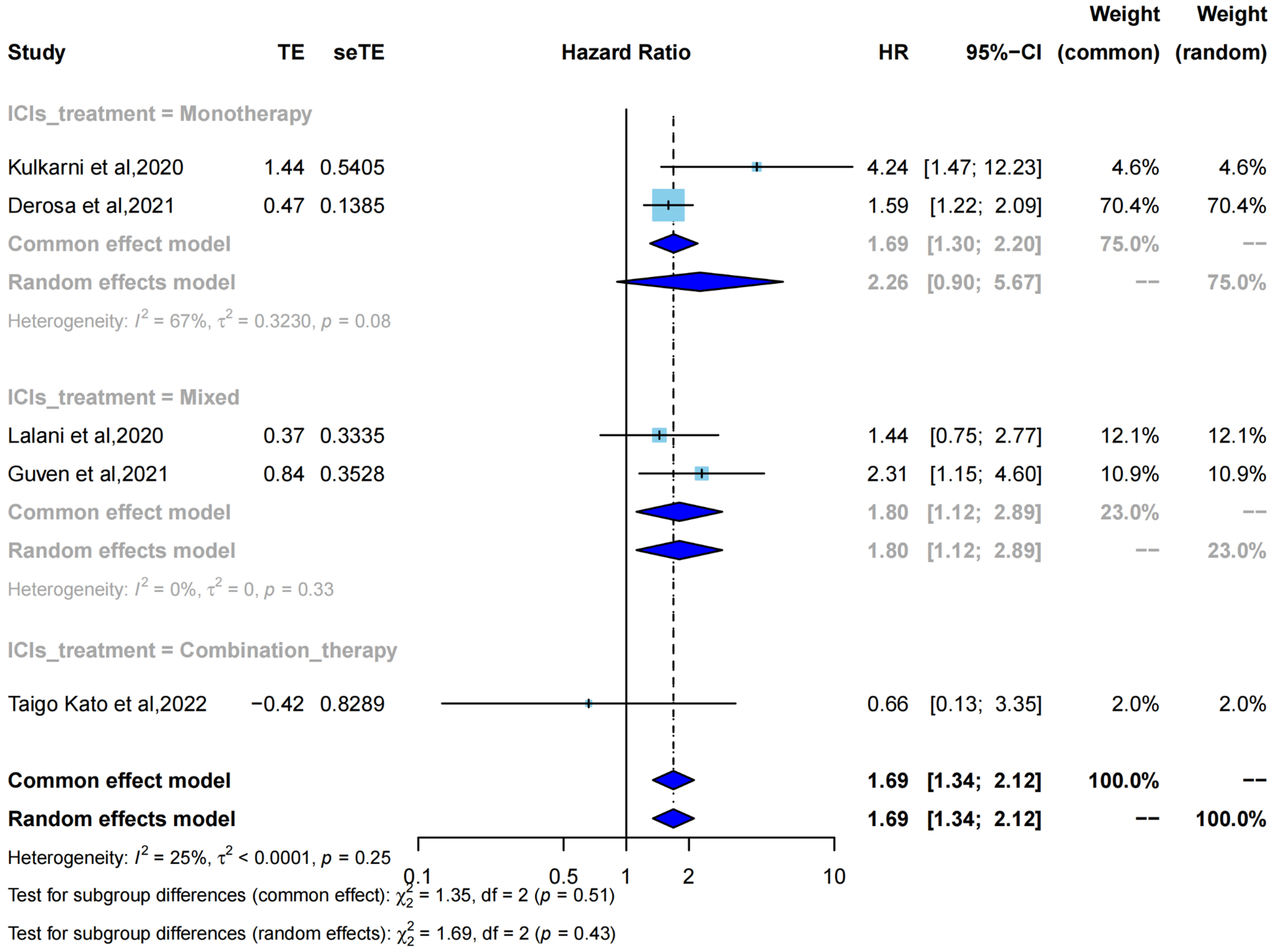

Supplement: Supplementary file 1 [file DataSheet_1.pdf]
